# HSF-1 is a regulator of miRNA expression in *Caenorhabditis elegans*

**DOI:** 10.1371/journal.pone.0183445

**Published:** 2017-08-24

**Authors:** Jessica Brunquell, Alana Snyder, Feng Cheng, Sandy D. Westerheide

**Affiliations:** 1 Department of Cell Biology, Microbiology, and Molecular Biology, College of Arts and Sciences, University of South Florida, Tampa, Florida, United States of America; 2 Department of Pharmaceutical Sciences, College of Pharmacy, University of South Florida, Tampa, Florida, United States of America; 3 Department of Biostatistics, College of Public Health, University of South Florida, Tampa, Florida, United States of America; Shanghai Ocean University, CHINA

## Abstract

The ability of an organism to sense and adapt to environmental stressors is essential for proteome maintenance and survival. The highly conserved heat shock response is a survival mechanism employed by all organisms, including the nematode *Caenorhabditis elegans*, upon exposure to environmental extremes. Transcriptional control of the metazoan heat shock response is mediated by the heat shock transcription factor HSF-1. In addition to regulating global stress-responsive genes to promote stress-resistance and survival, HSF-1 has recently been shown to regulate stress-independent functions in controlling development, metabolism, and longevity. However, the indirect role of HSF-1 in coordinating stress-dependent and -independent processes through post-transcriptional regulation is largely unknown. MicroRNAs (miRNAs) have emerged as a class of post-transcriptional regulators that control gene expression through translational repression or mRNA degradation. To determine the role of HSF-1 in regulating miRNA expression, we have performed high-throughput small RNA-sequencing in *C*. *elegans* grown in the presence and absence of *hsf-1* RNAi followed by treatment with or without heat shock. This has allowed us to uncover the miRNAs regulated by HSF-1 via heat-dependent and -independent mechanisms. Integrated miRNA/mRNA target-prediction analyses suggest HSF-1 as a post-transcriptional regulator of development, metabolism, and longevity through regulating miRNA expression. This provides new insight into the possible mechanism by which HSF-1 controls these processes. We have also uncovered oxidative stress response factors and insulin-like signaling factors as a common link between processes affected by HSF-1-regulated miRNAs in stress-dependent and -independent mechanisms, respectively. This may provide a role for miRNAs in regulating cross-talk between various stress responses. Our work therefore uncovers an interesting potential role for HSF-1 in post-transcriptionally controlling gene expression in *C*. *elegans*, and suggests a mechanism for cross-talk between stress responses.

## Introduction

The ability of an organism to adapt to proteotoxic stressors is essential for long-term survival. The mammalian heat shock factor HSF1 is a highly conserved transcription factor that protects against extreme environmental conditions through induction of the cytoprotective heat shock response (HSR) [1]. Activation of the HSR is triggered by proteotoxic stressors including heat, heavy metals, and infection [2]. Upon exposure to stress, HSF1 transcribes heat shock protein genes (*hsps*) which encode molecular chaperones that mediate the refolding or degradation of damaged proteins [3, 4]. HSF1 is thus an important mediator of the response to heat stress and proteome maintenance.

The *C*. *elegans* HSR is highly conserved and mediated by the HSF1 homolog HSF-1. Studies in *C*. *elegans* have demonstrated a role for HSF-1 outside of its classic role in regulating the HSR by demonstrating HSF-1 as a regulator of development, metabolism, and longevity [5–9]. HSF-1 has also been shown to be a regulator of global mRNA expression in both stress-dependent and -independent processes [7]. However, the post-transcriptional role for HSF-1 in controlling stress-dependent and -independent gene expression is largely unknown.

MicroRNAs (miRNAs) are a family of small, non-coding, and conserved RNA molecules that elicit complex mechanisms of genetic control through the post-transcriptional modulation of gene expression. The primary function of miRNAs is in the silencing of gene expression through complimentary base pairing to the 3’ UTR of target mRNAs leading to their degradation or translational repression [10, 11]. The regulation of gene expression by miRNAs is suggested to be vast. There are over 1,000 miRNAs in the human genome and over 250 miRNAs in the *C*. *elegans* genome, and many miRNAs are predicted to have hundreds of putative mRNA targets [12]. Thus, miRNAs are important post-transcriptional regulators of global gene expression in multiple organisms.

Studies in *C*. *elegans* have been useful for determining the biological outcomes associated with changes in miRNA expression [13]. For example, miRNAs have been shown to control various physiological process including the control of stress responses, development, and longevity, in the nematode [14–17]. Interestingly, these miRNA-regulated processes are also known to be regulated by HSF-1. However, the role of HSF-1 in coordinating miRNA expression to regulate these overlapping biological processes is unclear.

In this study, we examined the genome-wide role of HSF-1 in regulating miRNA expression to affect biological processes in *C*. *elegans*. We performed high-throughput small-RNA sequencing in *C*. *elegans* grown in the presence and absence of *hsf-1* RNAi followed by treatment with or without heat shock (HS). We have found that HSF-1 controls miRNA expression during and independently of heat stress. The biological processes predicted to be impacted by HSF-1-regulated miRNAs include development, metabolism, and longevity. Additionally, integrated miRNA/mRNA target prediction analyses have uncovered oxidative stress response factors and insulin-like signaling factors as a common link between processes affected by HSF-1 regulated miRNAs in stress-dependent and -independent mechanisms, respectively. Overall, this work highlights miRNAs as important HSF-1 targets that may have biological implications in regulating development, metabolism, and longevity, and in cross-talk between stress responses.

## Materials and methods

### *C*. *elegans* strains and maintenance

The wild-type N2 strain, p*hsp-70*(*C12C8*.*1*)::GFP [5], and p*hsp-16*.*2*(*Y46H3A*.*3*)::GFP [18] strains were maintained at 23°C on standard NGM plates seeded with *Escherichia coli* OP50 [19]. Age synchronization was accomplished by standard 20% hypochlorite treatment, and a 24-hour rotation at 220 rpm in M9 buffer without food.

### Fluorescence microscopy and quantification

Animals were anesthetized with 10 mM levamisole and photographed using an EVOS fluorescence microscope. Image processing was accomplished using Adobe Photoshop (Adobe Systems Incorporated, San Jose, CA). Quantification of fluorescence intensity was performed using ImageJ Software (v. 1.44; http://imagej.nih.gov/ij/).

### RNA interference and heat shock treatment

RNAi was carried out using standard plates supplemented with 50 μg/mL ampicillin and 1 mM isopropyl-beta-ᴅ-thiogalactopyranoside seeded with HT115 bacteria containing an empty plasmid (L4440, empty vector control) or sequence-verified *hsf-1* RNAi isolated from the Ahringer RNAi library [20]. Bacteria were allowed to induce double-stranded RNA on the RNAi plates overnight at room-temperature prior to plating synchronous L1 larval stage worms. Worms remained on RNAi plates until the L4 larval-stage before being heat shocked by submerging plates in a 33°C water bath for 30 minutes. The time and duration of heat shock was previously optimized for our studies [7].

### miRNA preparation for miRNA-seq

miRNA was prepared from biological duplicates using TRIzol reagent (Life Technologies, *cat*.*#* 15596–026) following the manufacturer’s protocol, and then cleaned up on miRNeasy columns (Qiagen, *cat*.*#* 217004) with on-column DNA digestion. Sample integrity, preparation, and sequencing was performed at the Yale Center for Genome Analysis (West Haven, CT) using the Illumina Hi-Seq 2000 sequencing system.

### miRNA-sequencing data analysis

The miRDeep2 software package was used to identify known miRNAs from the miRNA-sequencing data [21]. Low quality reads, and reads shorter than 18 nucleotides, were removed to obtain clean reads after adapter trimming. The unique sequences were mapped to the *C*. *elegans* reference genome (WS200) with the read aligner Bowtie2 using default parameters [22]. Alignments with no mismatches in the first 18 nucleotides of a read sequence, up to two mismatches after 18 nucleotides, and reads that did not map more than five times to the genome, were the miRNAs used in our analyses. The results were visualized with a dendogram using the program CummeRbund. Scatter plots were created for the miRNA-seq reads for each biological replicate, for each condition, to show similarity between replicates. The identified miRNAs that are significantly regulated by HSF-1 during and independently of HS all correspond to the 5p strand. The data sets supporting the results of this article are available in the NCBI SRA repository, accession number SRP097189.

### Volcano plot generation

Volcano plots were made in Excel, where the Y-axis represents the -log_10_ q-value (FDR-adjusted p-value), and the X-axis represents the log_2_-fold change of each miRNA after normalization to the *hsf-1*(+);-HS control.

### miRNA-seq data normalization and statistical analysis

The fold change of each miRNA was obtained by averaging the reads of the *hsf-1*(+);-HS control and normalizing each corresponding miRNA, for each treatment condition, to the control prior to transforming into log_2_ values. miRNAs that did not have reads in each biological replicate were removed from subsequent analyses. The Benjamini-Hochberg correction for multiple testing was then used to determine significantly altered miRNAs between each treatment condition.

### Venn diagram analysis

Venny 2.0 [23] was used to construct Venn diagrams with the significantly altered miRNAs for each condition (q-value<0.05) as compared to the *hsf-1*(+);-HS control.

### Quantitative RT-PCR

qRT-PCR was performed to validate hits from our miRNA-seq data using newly generated miRNA samples in biological duplicate and technical triplicate. cDNA synthesis was performed from the miRNA samples using the miScript II RT Kit (Qiagen, cat. # 218160) following the manufacturer’s instructions. For qRT-PCR, the miScript SYBR Green PCR Kit (Qiagen cat. # 218073) was utilized along with MiScript Primer Assays for the indicated miRNAs (Qiagen), following the manufacturer’s instructions and performed with the Step One Plus Real-time PCR system (Applied Biosystems). Data analysis was performed according to standard calculations using the comparative Ct method [24]. Relative miRNA levels were normalized to miR-2-5p, which was previously determined to have stable expression under various stress-induced conditions including heat shock [25].

### Computational target prediction and network visualization

mirWIP was used as a primary tool for determining miRNA/mRNA target predictions [26]. For miRNAs not yet listed in the mirWIP database, TargetScan was used to predicted miRNA/mRNA interactions [27]. To consolidate the predicted mRNA targets, the output from mirWIP or TargetScan was compared to corresponding mRNA-seq data, performed in parallel to this miRNA-seq experiment. The mRNA-seq data is available in the NCBI SRA database (Access ID: PRJNA311958) [7]. The network building software Agilent was then used to determine interacting partners shared between at least two predicted mRNA targets, and the miRNA/mRNA interaction network was generated using Cytoscape (v3.1.1) [28].

### Gene ontology analysis

The Database for Annotation, Visualization, and Integrated Discovery (DAVID) was used to determine over-represented gene ontology terms associated with the predicted miRNA/mRNA networks [29]. The Functional Annotation Clustering tool was then used to group gene ontology terms. The significance of each group was determined with the enrichment score, as provided by DAVID, which uses p-values for a cluster of genes to determine the geometric mean of that cluster in a negative log scale.

## Results

### Uncovering the genome-wide regulation of miRNA expression by HSF-1 in HS-dependent and -independent mechanisms

#### Experimental design used for miRNA-sequencing

**Fig 1:**
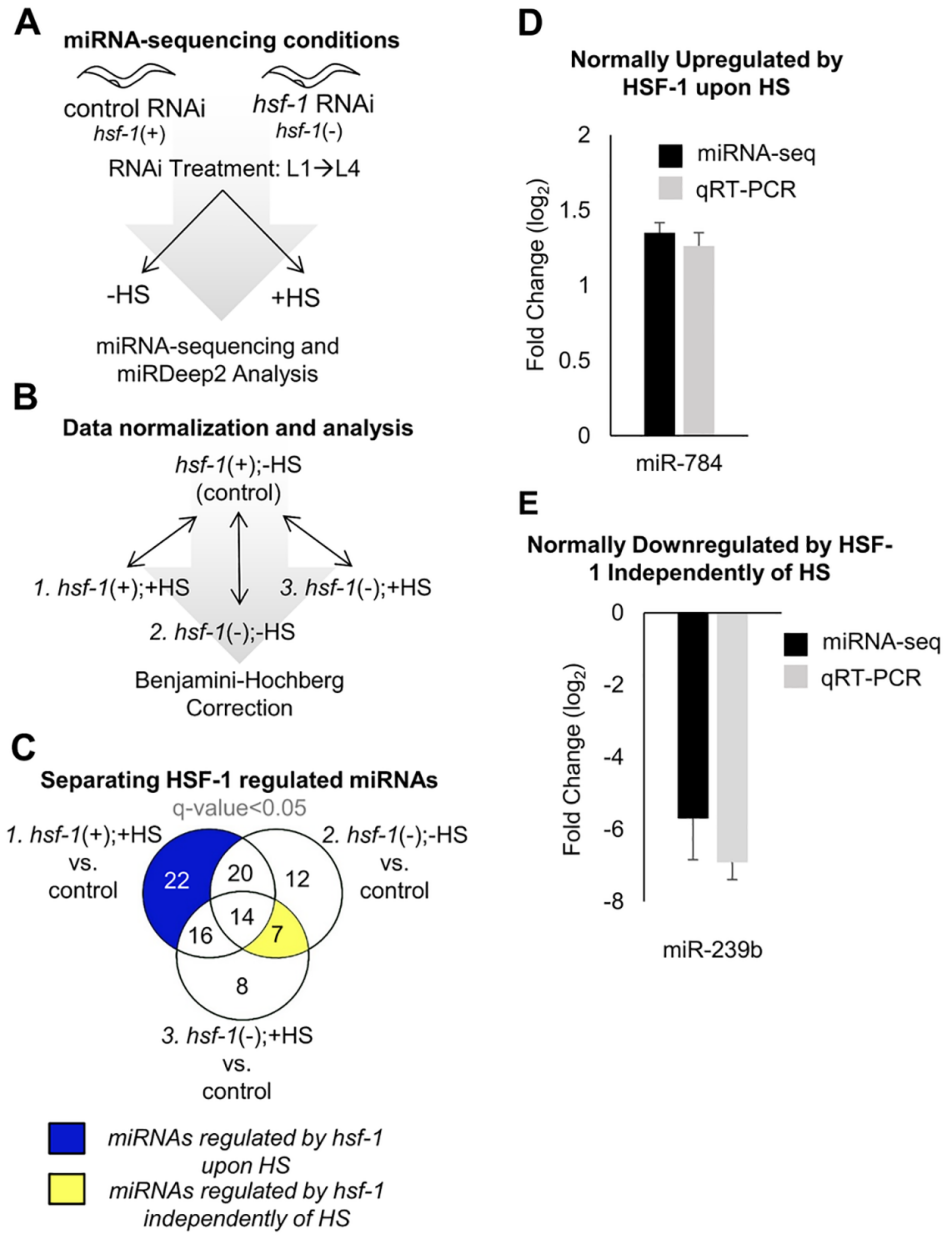
Scheme for miRNA-sequencing experimental setup and data normalization. (**A**) Schematic depicting miRNA-sequencing conditions. miRNA samples were generated from wildtype (N2) *C*. *elegans* treated with the four indicated conditions, where “*hsf-1*(+)” refers to worms treated with control (empty vector) RNAi, and “*hsf-1*(-)” refers to worms treated with *hsf-1* RNAi. Synchronous worms were given RNAi from the L1 larval stage to the L4 larval stage. At the L4 larval stage, worms were left untreated (-HS) or given a 30 minute 33°C heat shock (+HS). miRNA-sequencing was performed in biological duplicates on the Illumina Hi-Seq 2000 platform and analyzed using miRDeep2. (**B**) Scheme for data normalization. Each treatment condition was compared relative to the empty vector control [*hsf-1*(+);-HS] to determine the relative fold change in expression of each miRNA. The Benjamini-Hochberg correction test was used to identify all differentially expressed genes relative to the *hsf-1*(+);-HS control, and also between treatment conditions. (**C**) Separating miRNAs regulated by HSF-1 during and independently of HS. The Venn diagram shows the total number of miRNAs that were found to be significantly altered (q-value<0.05), as compared to the *hsf-1*(+);-HS control, for each of the indicated comparisons between samples. The q-value is the FDR-adjusted p-value of the test statistic, as determined by the Benjamini-Hochberg correction for multiple testing. (**D**) Validation of miRNA-seq hits for HSF-1-regulated miRNAs. The log_2_ fold change of miR-784, a miRNA normally upregulated by HSF-1 upon HS, in the *hsf-1*(+); +HS vs control condition. (**E**) The log_2_ fold change of miR-239b, a miRNA normally downregulated by HSF-1 independently of HS, in the *hsf-1*(-);-HS vs control condition.

To uncover the post-transcriptional role of HSF-1 in regulating mRNA expression through controlling miRNA abundance, we utilized miRNA-sequencing to characterize the HSF-1 regulatory miRNA network in both HS-dependent and -independent mechanisms in *C*. *elegans* (Fig 1). Synchronous L1 larval stage nematodes were fed empty vector (EV) control RNAi [*hsf-1*(+)] or *hsf-1* RNAi [*hsf-1*(-)] until the L4 larval stage prior to treatment with or without a 30 minute 33°C heat shock in biological duplicates (Fig 1A). miRNA-seq was performed on the Illumina Hi-Seq 2000 platform and analyzed by miRDeep2 in order to identify miRNA read counts for each treatment condition. This experimental set-up thus allows for the determination of the genome-wide miRNAs regulated by HSF-1.

#### Validation of experimental treatment conditions

We have validated our RNAi and HS treatment conditions in a number of ways. First, in a mRNA-seq study that was performed in parallel to this one [7], we validated the efficacy of *hsf-1* RNAi by qRT-PCR, We now also assess HS-inducible *hsp* promoter activity and mRNA expression in response to treatment with or without *hsf-1* RNAi (S1 Fig) [7]. Additionally, the promoter activity of the HSF-1 target genes *hsp-70* and *hsp-16*.*2* were determined using *hsp* promoter fusion constructs, and we have found that *hsp* promoter activity is induced upon HS and that this induction is dependent on HSF-1 (S1A–S1D Fig). *hsps* constitute a majority of the top protein-coding genes induced by HS, and all are dependent on HSF-1, as determined by an mRNA-seq experiment performed in parallel to this miRNA-seq study (S1E Fig) [7]. These data thus verify that the HS conditions used for this study induce global *hsp* mRNA expression while demonstrating the efficiency of our *hsf-1* RNAi treatment conditions used for miRNA-seq.

Next, we validated the biological replicates used for miRNA-seq by determining similarities between experimental duplicates for each experimental treatment condition (S2 Fig). Similarities between biological duplicates were first analyzed with Cluster analysis. The data shows that experimental duplicates clustered together, indicating high similarity between replicates (S2A Fig). Linear regression analysis of each treatment condition validates similarities between miRNA-seq reads for each replicate, with each condition having R^2^ values above 0.94 (S2B–S2E Fig). Overall, these data validate our biological replicates by showing a conserved pattern of expression between duplicates for each experimental condition.

#### HSF-1 alters global miRNA abundance during and independently of HS

To determine relative miRNA abundance, we normalized each treatment condition to the *hsf-1*(+);-HS control, and significantly altered miRNAs were uncovered using the Benjamini-Hochberg correction for multiple testing (Fig 1B). A complete list of the significantly altered miRNAs, and their fold change for each experimental condition relative to the *hsf-1*(+);-HS control, is provided in S1 Table. The resulting global miRNA expression profiles for each treatment condition were plotted in order to visualize miRNA distribution patterns between experimental conditions (S3 Fig). In the presence and absence of HS, *hsf-1* RNAi alters global miRNA distribution, suggesting HSF-1 as a regulator of miRNA expression during and independently of HS in *C*. *elegans*.

#### Venn diagram analyses separate miRNAs regulated by HSF-1 during and independently of HS

To further examine the role of HSF-1 in regulating miRNA expression in HS-dependent and HS-independent mechanisms, Venn diagrams were made using the miRNAs listed in S1 Table (Fig 1C). The shaded regions of the Venn diagrams correspond to miRNAs regulated by *hsf-1* upon HS (Fig 1C, blue shading), and miRNAs regulated by *hsf-1* independently of HS (Fig 1C, yellow shading). Through Venn diagram analysis, we have found that a total of 22 miRNAs are regulated by HSF-1 in a HS-dependent manner, and that 7 miRNAs are regulated by HSF-1 in a HS-independent manner. Each miRNA that we found to be significantly regulated by HSF-1 during and independently of HS corresponds to the 5p strand. A complete list of the miRNAs found in each section of the Venn diagram is provided in S2 Table. To validate our miRNA-seq data, we performed qRT-PCR with independent samples for miR-784 and miR-239b, two miRNA genes identified to have altered HSF-1-dependent expression, and found that similar results were obtained (Fig 1D and 1E). Overall, we have uncovered miRNAs regulated by HSF-1 during and independently of heat stress, and our following analyses focus on these HSF-1-regulated miRNAs.

### HSF-1 regulates miRNA expression during HS

#### miRNAs normally upregulated by HSF-1 during HS

**Fig 2:**
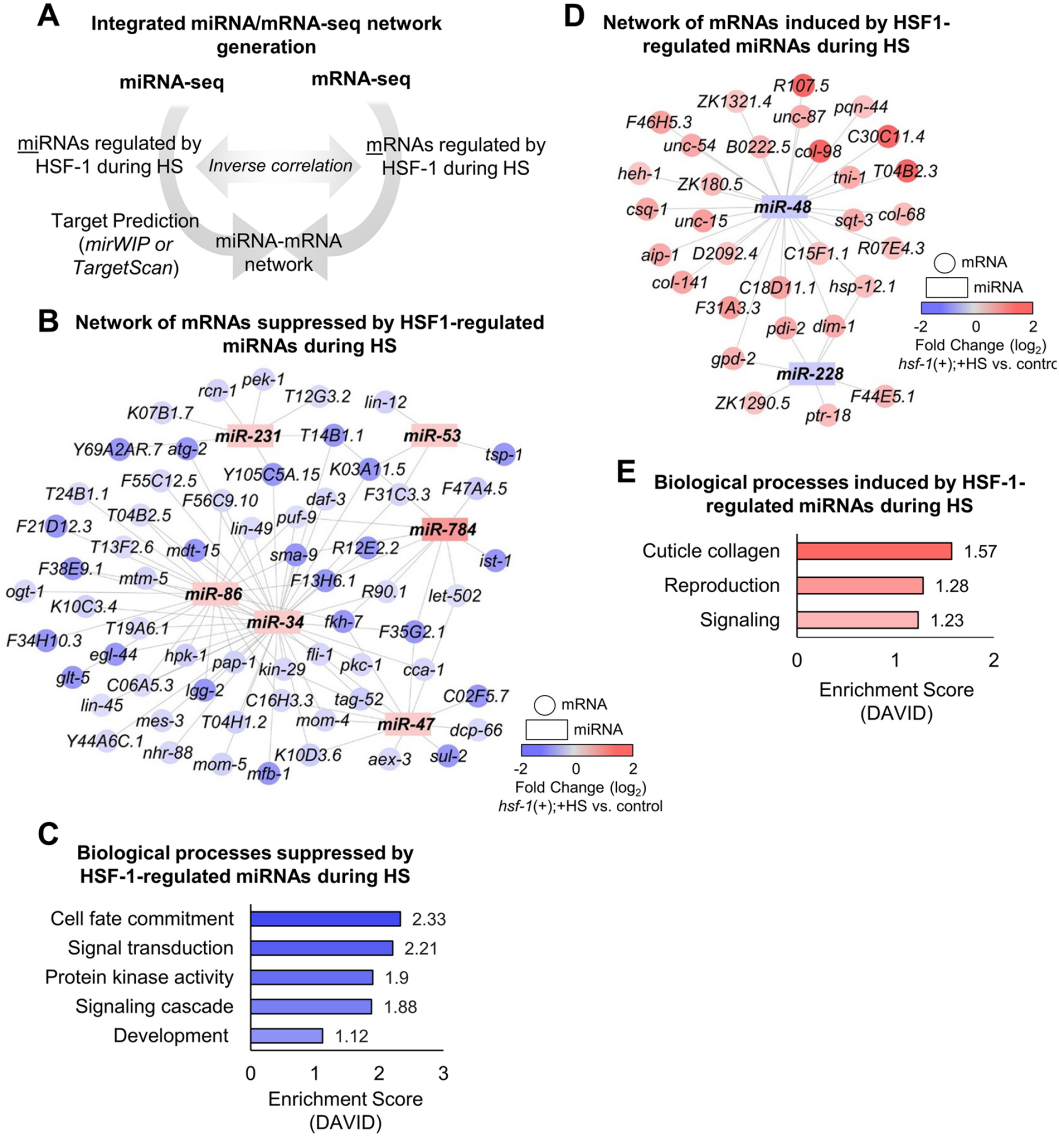
Networks and biological processes impacted by HSF-1-regulated miRNAs during HS. (**A**) Schematic depicting integrated miRNA/mRNA network generation. The miRNAs determined via miRNA-seq to be regulated by HSF-1 during HS were run through the target prediction tools mirWIP or TargetScan to uncover predicted mRNA targets. Due to the inhibitory nature of miRNAs, predicted mRNA targets were inversely correlated to mRNAs found to be regulated by HSF-1 during HS via mRNA-seq performed in parallel to this study [7]. (**B**) Predicted network suppressed by HSF-1-regulated miRNAs upon HS. The miRNAs that we found to normally be induced by HSF-1 during HS via miRNA-seq (rectangles) were compared to mRNAs previously determined to normally be suppressed by HSF-1 during HS via mRNA-seq (circles). (**C**) Biological processes predicted to be suppressed by HSF-1-regulated miRNAs during HS. DAVID was used to uncover biological processes predicted to be suppressed by HSF-1 upon HS using the network in (B). (**D**) Predicted network induced by HSF-1-regulated miRNAs upon HS. The miRNAs that we found to normally be suppressed by HSF-1 during HS via miRNA-seq (rectangles) were compared to mRNAs previously determined to normally be induced by HSF-1 via mRNA-seq (circles). (**E**) Biological processes predicted to be induced by HSF-1-regulated miRNAs during HS. DAVID was used to uncover biological processes predicted to be induced by HSF-1 upon HS using the network in (D).

We were next interested in further examining the miRNAs normally regulated by HSF-1 during HS (Fig 2). By separating out the upregulated vs. downregulated miRNAs via Venn diagram analysis, we have determined that 10 miRNAs are normally upregulated by HSF-1 upon HS (S4A Fig, blue shading). The log_2_-fold changes of these 10 miRNAs obtained from the sequencing data are plotted in the presence and absence of HSF-1 during HS (S4B Fig, blue and grey bars, respectively). We considered a miRNA to normally be upregulated by HSF-1 during HS if a significant difference existed between the *hsf-1*(+);+HS and *hsf-1*(-);+HS treatment conditions as determined by the Benjamini-Hochberg correction for multiple testing. The complete output from the Benjamini-Hochberg correction test can be found in S3 Table. After taking these parameters into account, 6 miRNAs were determined to normally be upregulated by HSF-1 during HS (S4C Fig). Through this method of data analysis, we found that *miR-784*, *miR-231*, *miR-86*, *miR-53*, *miR-47*, and *miR-34* are normally upregulated by HSF-1 during HS.

Next, we determined the known functions of the miRNAs we found to normally be upregulated by HSF-1 upon HS. The description according to WormBase, along with the log_2_-fold change of each miRNA, is listed (Table 1, upregulated). While the functions of *miR-784*, *miR-231*, *miR-86*, *miR-53*, and *miR-47* are currently unknown, *miR-34* encodes a microRNA that is highly conserved with orthologues in *Drosophila*, mouse, and human [30]. In *C*. *elegans*, *miR-34* is highly expressed upon aging [14]. Although a deletion of *miR-34* does not significantly impact *C*. *elegans* lifespan [14], loss of *miR-34* in *Drosophila* does accelerate aging and brain degeneration [30]. Induction of *miR-34* by HSF-1 during HS may thus be one method of controlling aging-associated processes.

**Table 1:** miRNAs normally regulated by HSF-1 upon HS.

miRNA	Fold Change (log_2_) *hsf-1*(+);+HS vs. control	Description (WormBase)
**Normally upregulated by *hsf-1* upon heat shock**		
*miR-784*	1.35	*miR-784* is expressed in head neurons and the vulva, however the precise function of *miR-784* is unknown.
*miR-231*	0.67	*miR-231* may have a potential ortholog in *C*. *briggsae*. *miR-231* is strongly expressed at all stages of development in wild-type worms, however the precise function of *miR-231* is unknown.
*miR-86*	0.58	*miR-86* is conserved in the nematode *C*. *briggsae*. *mir-86* is strongly expressed at all developmental stages in wild-type worms, however the precise function of *miR-86* is unknown.
*miR-53*	0.38	*miR-53* is expressed constitutively throughout development, however the precise function is unknown.
*miR-47*	0.22	*miR-47* is conserved in *C*. *briggsae*. *mir-47* is expressed constitutively throughout development, however the precise function of *miR-47* is unknown.
*miR-34*	0.15	*miR-34* is conserved in *C*. *briggsae*, *Drosophila*, and humans. *mir-34* can regulate adult lifespan along with resistance to heat and oxidative stress. *miR-34* functions via negative regulation of autophagy.
**Normally downregulated by *hsf-1* upon heat shock**		
*miR-228*	-0.13	*miR-228* appears to be conserved in *C*. *briggsae*. *miR-228* belongs to the *miR-124* family of microRNAs along with human *miR-124a-1*, *miR-124a-2*, *miR-124-a-3*, m*iR-183*, and *Drosophila miR-268*. *miR-228* is upregulated during aging, and a deletion of *miR-228* increases longevity and stress resistance.
*miR-48*	-0.13	*miR-48* belongs to the *let-7* family of microRNAs. *miR-48* can act with other *let-7* family members to control developmental timing events.

The log_2_-fold change of each miRNA is listed along with a description adapted from WormBase.

To uncover potential mRNA targets of the miRNAs we found to be regulated by HSF-1 upon HS, we performed integrated prediction-based analysis by comparing predicted mRNA targets to genes we previously determined to be regulated by HSF-1 upon HS via mRNA-seq (Fig 2A) [7]. We used mirWIP as our primary prediction tool, as this program reduces false positives by considering multiple characteristics of miRNA target binding such as structural accessibility of target sequences, total free energy, and base-pairing [26]. For miRNAs not yet in the mirWIP database, we used TargetScan to predict mRNA targets. In order to generate a consolidated and accurate HSF-1-regulated miRNA/mRNA network, we compared the predicted mRNA targets to genes we previously determined by mRNA-seq, performed in parallel to this study, to be regulated by HSF-1 [7]. Due to the inhibitory nature of miRNAs on target mRNA expression, predicted mRNA targets were inversely correlated to the mRNA-seq data. The resulting integrated miRNA/mRNA prediction output anticipated to be suppressed by HSF-1 upon HS is shown in Fig 2B. Overall, 62 potential mRNA targets are predicted to be downregulated by the miRNAs that we determined to normally be upregulated by HSF-1 upon HS.

Next, we used the database for annotation, visualization, and integrated discovery (DAVID) to uncover the biological processes predicted to be suppressed by the miRNAs upregulated by HSF-1 during HS (Fig 2C). The category with the largest enrichment score (2.33) is cell fate commitment, followed by signal transduction, protein kinase activity, signaling cascade, and development, all with enrichment scores between 2.21–1.12. These processes encompass developmental and signaling pathways, which are likely the main physiological processes suppressed by HSF-1-regulated miRNAs during HS.

#### miRNAs normally downregulated by HSF-1 during HS

We were next interested in examining the miRNAs normally downregulated by HSF-1 during HS (Fig 2D and 2E). By separating out the upregulated vs. downregulated miRNAs via Venn diagram analysis, we determined that 12 miRNAs are normally downregulated by HSF-1 upon HS (S5A Fig, blue shading). The log_2_-fold change of these 12 miRNAs are plotted in the presence and absence of HSF-1 during HS (S5B Fig, blue bars and grey bars, respectively). We considered a miRNA to normally be downregulated by HSF-1 during HS if a significant difference existed between the *hsf-1*(+);+HS and *hsf-1*(-);+HS treatment conditions as determined by the Benjamini-Hochberg correction for multiple testing. The complete output from the Benjamini-Hochberg correction test can be found in S3 Table. After taking these parameters into account, 2 miRNAs were determined to normally be downregulated by HSF-1 during HS (S5C Fig). Through this method of data analysis, we found that *miR-48* and *miR-228* are normally downregulated by HSF-1 upon HS.

We were next interested in determining the known functions of the miRNAs we found to normally be downregulated by HSF-1 upon HS. The description according to WormBase, along with the log_2_-fold change of each miRNA, is listed (Table 1, downregulated). *miR-228* is upregulated in aging worms, and a *miR-228* deletion has been shown to increase longevity and heat stress resistance [15, 31]. *miR-48* belongs to the *let-7* miRNA family that is well-known to control developmental timing events at the larval-to-adult transition, and mutations in *miR-48* can result in developmental timing defects [32, 33]. Thus, HSF-1 may normally suppress *miR-228* and *miR-48* expression during HS to promote longevity and stress resistance, and to control developmental timing events during stress conditions.

To uncover potential mRNA targets of the miRNAs we found to be downregulated by HSF-1 upon HS, we again used the prediction tools mirWIP and TargetScan followed by an integrated miRNA/mRNA analysis (Fig 2D). We compared the predicted mRNA targets to genes we previously determined by mRNA-seq, performed in parallel to this study, to be upregulated by HSF-1 during HS [7]. Overall, 31 potential mRNA targets are predicted to be upregulated by the miRNAs that we determined to normally be downregulated by HSF-1 upon HS.

Next, we used DAVID to uncover the biological processes predicted to be induced by HSF-1-regulated miRNAs during HS (Fig 2E). The category with the largest enrichment score (1.57) is cuticle collagen, followed by reproduction and signaling, with enrichment scores of 1.28 and 1.23, respectively. These processes encompass aging and signaling associated pathways, which are likely the main physiological processes induced by HSF-1-regulated miRNAs during HS.

### HSF-1 regulates miRNA expression independently of HS

#### miRNAs normally upregulated by HSF-1 independently of HS

**Fig 3:**
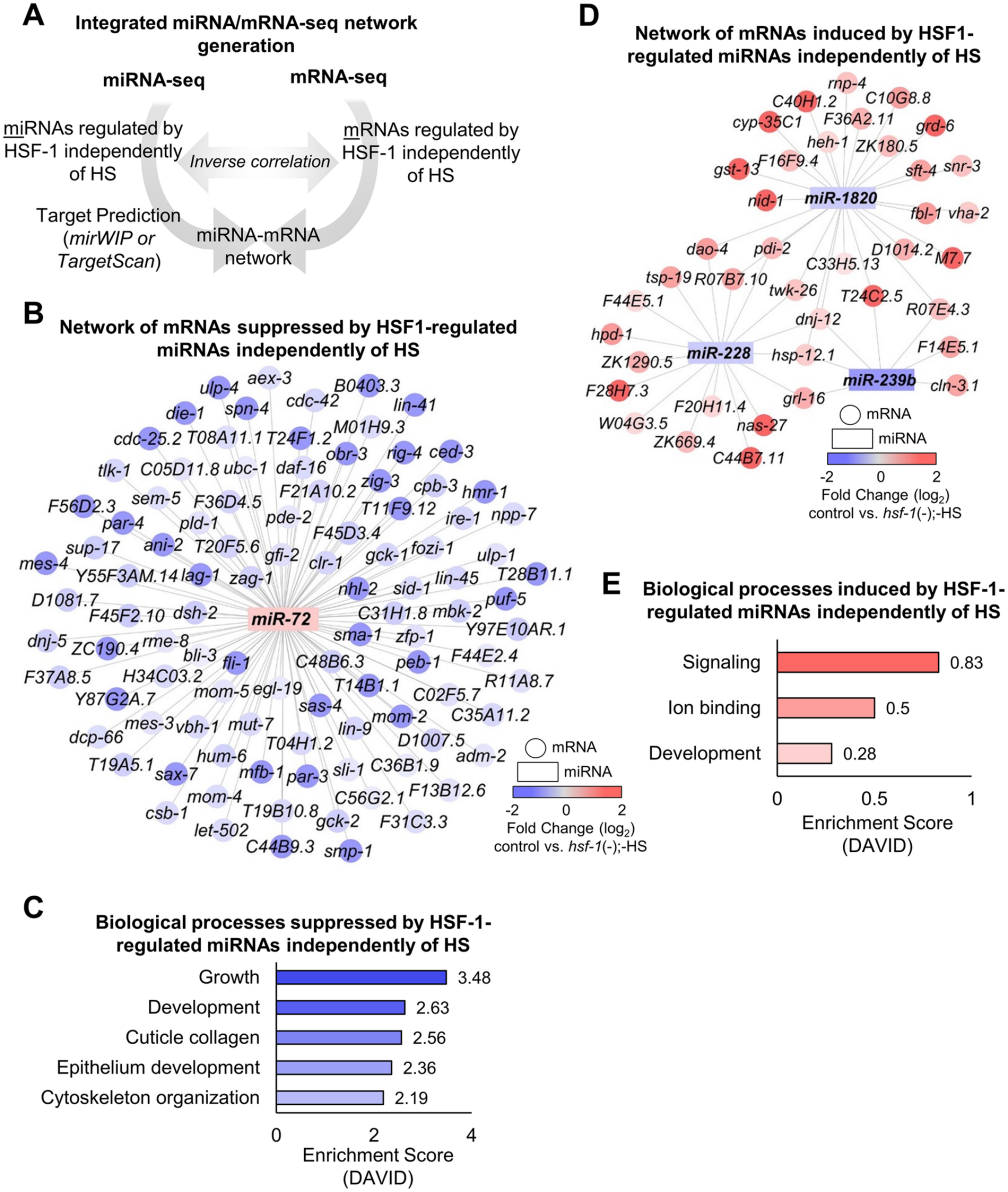
Networks and biological processes impacted by HSF-1-regulated miRNAs independently of HS. (**A**) Schematic depicting integrated miRNA/mRNA network generation. The miRNAs determined via miRNA-seq to be regulated by HSF-1 independently of HS were run through the target prediction tools mirWIP or TargetScan to uncover predicted mRNA targets. Due to the inhibitory nature of miRNAs, predicted mRNA targets were inversely correlated to mRNAs found to be regulated by HSF-1 independently of HS via mRNA-seq performed in parallel to this study [7]. (**B**) Predicted network suppressed by HSF-1-regulated miRNAs independently of HS. The miRNAs that we found to normally be induced by HSF-1 independently of HS via miRNA-seq (rectangles) were compared to mRNAs previously determined to normally be suppressed by HSF-1 independently of HS via mRNA-seq (circles). (**C**) Biological processes predicted to be suppressed by HSF-1-regulated miRNAs independently of HS. DAVID was used to uncover biological processes predicted to be suppressed by HSF-1 independently of HS using the network in (B). (**D**) Predicted network induced by HSF-1-regulated miRNAs independently of HS. The miRNAs that we found to normally be suppressed by HSF-1 independently of HS via miRNA-seq (rectangles) were compared to mRNAs previously determined to normally be induced by HSF-1 independently of HS via mRNA-seq (circles). (**E**) Biological processes predicted to be induced by HSF-1-regulated miRNAs independently of HS. DAVID was used to uncover biological processes predicted to be induced by HSF-1 independently of HS using the network in (D).

To uncover a HS-independent role for HSF-1 in regulating miRNA expression, we next focused on the miRNAs normally regulated by HSF-1 independently of HS (Fig 3). By separating out the upregulated vs. downregulated miRNAs via Venn diagram analysis, we determined that 2 miRNAs are downregulated in response to *hsf-1* RNAi independently of HS, thus these miRNAs would normally be upregulated by HSF-1 (S6A Fig, yellow shading). The log_2_-fold change of these 2 miRNAs are plotted in the absence of HSF-1 and in the presence and absence of HS (S6B Fig, grey and yellow bars, respectively). To determine the normal regulatory role of HSF-1 on miRNA expression, we reversed our data comparison (control vs. indicated treatment conditions). We considered a miRNA to be regulated by HSF-1 independently of HS if no significant difference existed between the *hsf-1*(-);+HS and *hsf-1*(-);-HS treatment conditions using the Benjamini-Hochberg correction for multiple testing. The complete results from the Benjamini-Hochberg correction test can be found in S3 Table. After taking these parameters into account, 1 miRNA was determined to normally be upregulated by HSF-1 independently of HS (S6C Fig). Through this method of data analysis, we found that *miR-72* is normally upregulated by HSF-1 independently of HS.

Next, we determined the known functions of the miRNA we found to normally be upregulated by HSF-1 independently of HS. The description according to WormBase, along with the log_2_-fold change of each miRNA, is listed (Table 2, upregulated). We again reversed our data comparison [control vs. *hsf-1*(-);-HS] in order to represent the normal degree of HSF-1 regulation on miRNA expression. However, the function of *miR-72* is unknown, thus warranting future studies regarding *miR-72*.

**Table 2:** miRNAs normally regulated by HSF-1 independently of HS.

miRNA	Fold Change (log_2_) control vs. *hsf-1*(-);-HS	Description (WormBase)
**Normally upregulated by *hsf-1* independently of heat shock**		
*miR-72*	0.12	*miR-72* is a member of the *mir-31* microRNA family that includes human *miR-31*. However, the precise function of *miR-72* is unknown.
**Normally downregulated by *hsf-1* independently of heat shock**		
*miR-239b*	-5.70	*miR-239b* is highly upregulated during aging. Deletion of *miR-239* can result in lifespan extension, while overexpression can lead to a reduction in lifespan.
*miR-1820*	-0.57	*mir-1820* is upregulated in dauer worms.
*miR-228*	-0.11	*miR-228* appears to be conserved in *C*. *briggsae*. *miR-228* belongs to the *miR-124* family of microRNAs along with human *miR-124a-1*, *miR-124a-2*, *miR-124-a-3*, m*iR-183*, and *Drosophila miR-268*. *miR-228* is upregulated during aging, and a deletion of *miR-228* increases longevity and stress resistance.

The log_2_-fold change of each miRNA is listed along with a description adapted from WormBase.

To uncover potential mRNA targets of the miRNAs regulated by HSF-1 independently of HS, we performed integrated prediction-based analysis by comparing predicted mRNA targets to genes we previously determined to be regulated by HSF-1 independently of HS via mRNA-seq (Fig 3A) [7]. Due to the inhibitory nature of miRNAs on target mRNA expression, predicted mRNA targets were inversely correlated to the mRNA-seq data. The resulting integrated miRNA/mRNA prediction output predicted to be suppressed by HSF-1 independently of HS is shown in Fig 3B. Overall, 101 potential mRNA targets are predicted to be downregulated by HSF-1-regulated miRNAs independently of HS.

We next used DAVID to uncover the biological processes predicted to be suppressed by HSF-1-regulated miRNAs independently of HS (Fig 3C). The category with the largest enrichment score (3.48) is growth, followed by development, cuticle collagen, epithelium development, and cytoskeleton organization with enrichment scores between 2.63–2.19. These processes all contribute to development, which is likely the main process suppressed by HSF-1-regulated miRNAs independently of HS.

#### miRNAs normally downregulated by HSF-1 independently of HS

We were next interested in determining a HS-independent role for HSF-1 in downregulating miRNA expression (Fig 3D and 3E). By separating out the upregulated vs. downregulated miRNAs via Venn diagram analysis, we have determined that 5 miRNAs are upregulated in response to *hsf-1* RNAi independently of HS, thus these miRNAs would normally be downregulated by HSF-1 (S6D Fig, yellow shading). The log_2_-fold change of these 5 miRNAs are plotted in the absence of HSF-1 and in the presence and absence of HS (S6E Fig, grey and yellow bars, respectively). To determine the normal regulatory role of HSF-1 on miRNA expression, we reversed our data comparison (control vs. indicated treatment conditions). We considered a miRNA to be regulated by HSF-1 independently of HS if no significant difference existed between the *hsf-1*(-);+HS and *hsf-1*(-);-HS treatment conditions using the Benjamini-Hochberg correction for multiple testing. The complete results from the Benjamini-Hochberg correction test can be found in S3 Table. After taking these parameters into account, 3 miRNAs were determined to normally be downregulated by HSF-1 independently of HS (S6F Fig). Through this method of data analysis, we have determined that *miR-239b*, *miR-1820*, and *miR-228* are normally downregulated by HSF-1 independently of HS.

Next, we determined the known functions of the miRNAs we found to normally be downregulated by HSF-1 independently of HS. The description according to WormBase, along with the log_2_-fold change of each miRNA, is listed (Table 2, downregulated). We again reversed our data comparison [control vs. *hsf-1*(-);-HS] in order to represent the normal degree of HSF-1 regulation. *miR-239b* is known to be highly upregulated upon aging, and mutants lacking *miR-239a/b* exhibit an increased lifespan and resistance to stress [14, 34]. One mechanism utilized by *miR-239b* to control longevity is through regulation of the insulin-like signaling pathway. *miR-239b* functions upstream of *daf-16* to control the expression of the *daf-16* target genes *age-1* and *pdk-1* [14]. Another miRNA we found to normally be downregulated by HSF-1 independently of HS is *miR-1820*. Although the function of *miR-1820* is currently unknown, dauer worms show increased expression of *miR-1820* suggesting a possible link to metabolism and longevity [35]. *miR-228* is another miRNA downregulated by HSF-1 independently of HS, however this miRNA is also downregulated by HSF-1 during HS. *miR-228* is thus likely an important HSF-1 target that may be regulated by HSF-1 to regulate longevity in both a stress-dependent and -independent fashion. Overall, we have determined that HSF-1 normally suppresses *miR-239b*, *miR-1820*, and *miR-228*, which may regulate longevity and metabolic processes independently of HS. To uncover potential mRNA targets of the miRNAs downregulated by HSF-1 independently of HS, we used the prediction tools mirWIP and TargetScan followed by integrated miRNA/mRNA analysis (Fig 3D). We compared the predicted mRNA targets to genes we previously determined by mRNA-seq, performed in parallel to this study, to be upregulated by HSF-1 independently of HS [7]. Overall, 39 potential mRNA targets are predicted to be induced by HSF-1-regulated miRNAs independently of HS.

We next used DAVID to uncover the biological processes predicted to be induced by HSF-1-regulated miRNAs independently of HS (Fig 3E). The category with the largest enrichment score (0.83) is signaling, followed by ion binding and development, with enrichment scores between 0.5–0.28. These processes encompass developmental and signaling processes, which are likely the main processes induced by HSF-1-regulated miRNAs independently of HS.

## Discussion

### HSF-1 may post-transcriptionally regulate gene expression by controlling miRNA abundance

miRNAs are emerging as a group of post-transcriptional modulators of gene expression that often function through translational repression or mRNA degradation. We show here that HSF-1 controls the expression of miRNAs, suggesting a post-transcriptional role for HSF-1 in regulating gene expression. Interestingly, the number of mRNAs predicted to be post-transcriptionally suppressed by HSF-1 during HS is twice as large as those predicted to be induced by HSF-1 during HS. Similarly, the number of mRNAs predicted to be post-transcriptionally suppressed by HSF-1 independently of HS is three times as large as those predicted to be induced by HSF-1 independently of HS. Overall, these data suggest that miRNAs may primarily be utilized by HSF-1 to suppress gene expression.

### Global biological processes impacted during HS by HSF-1-regulated miRNAs

#### Oxidative stress response factors link miRNA/mRNA networks regulated by HSF-1 during HS

**Fig 4:**
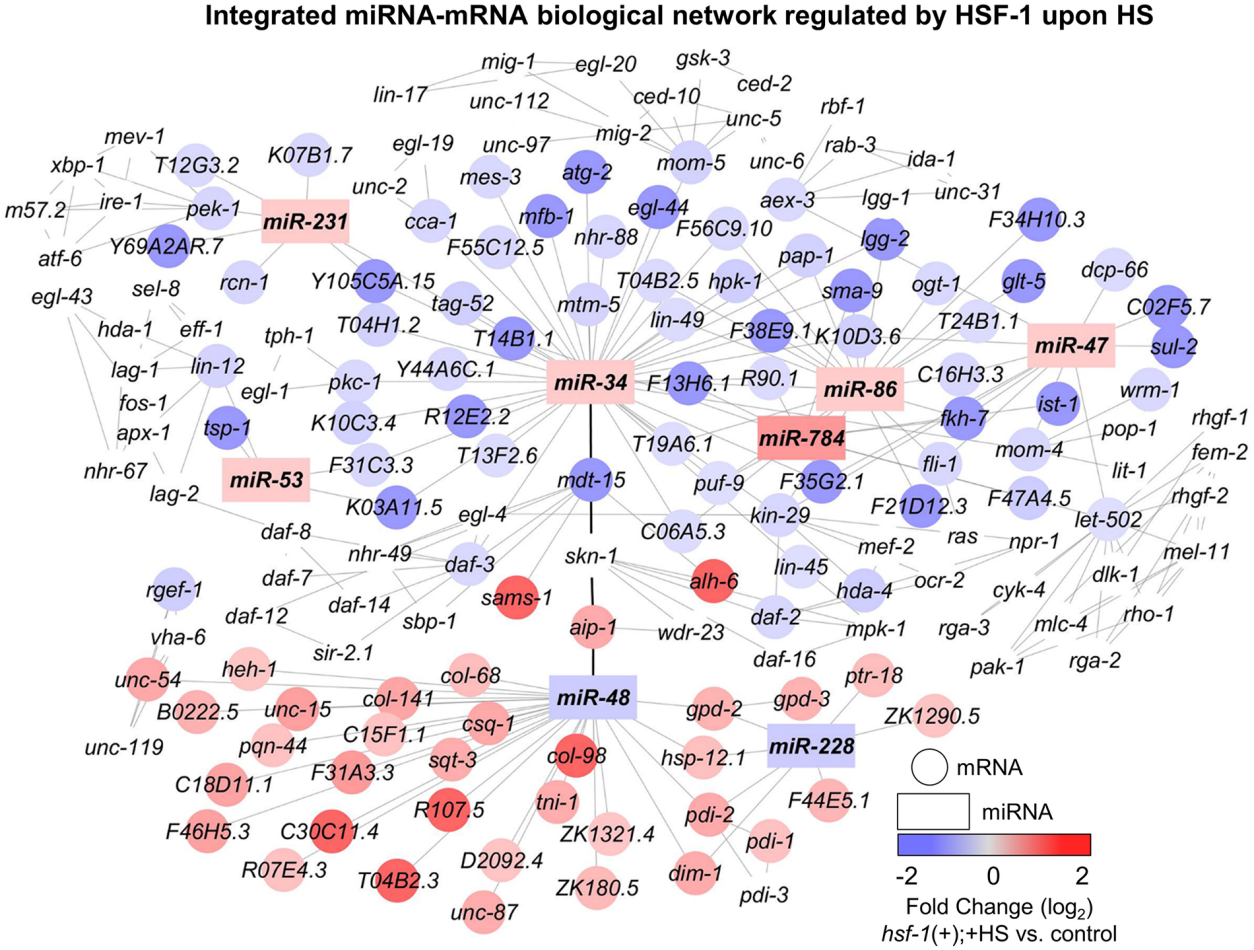
Integrated target prediction analysis uncovers miRNA/mRNA interaction networks regulated by HSF-1 during HS. The miRNAs (rectangles) that we determined to be regulated by HSF-1 during HS were used for target prediction analysis carried out by mirWIP or TargetScan. The mRNA targets (circles) were consolidated by comparing the predicted mRNAs to those determined by mRNA-sequencing, performed in parallel to miRNA sequencing, to be regulated by HSF-1 during HS. Interactions were predicted using the Agilent literature search tool, and network generation was done with Cytoscape. Transcripts that are not colored were not affected in our dataset, but are neighbors shared by at least two transcripts that were affected. The color of each miRNA or mRNA corresponds to the degree of HSF-1 regulation, where red represents induction and blue represents suppression. Bold connecting lines represent connections between upregulated and downregulated clusters.

To determine a broad impact for HSF-1 in post-transcriptionally regulating gene expression during HS, the network-building software Cytoscape and the Agilent literature search tool were used to build an interaction network with the integrated miRNA/mRNA network that we determined to be regulated by HSF-1 upon HS (Fig 4). Predicted mRNA targets were overlaid with the expression changes obtained from mRNA-seq, performed in parallel to this study [7], where blue corresponds to negative regulation and red corresponds to positive regulation by HSF-1 upon HS. Uncolored mRNA genes were not affected in our dataset but are neighbors shared by at least two genes that were affected. This method of network generation thus allows for the determination of a broad network of genes predicted to be post-transcriptionally regulated by HSF-1 during HS.

To uncover important links between the induced and suppressed networks, we grouped together the upregulated and downregulated interaction groups to visualize connecting mRNAs (Fig 4, represented as bold connecting lines). We have identified the oxidative stress response factors *mdt-15*, *skn-1*, and *aip-1* as links between the induced and suppressed networks regulated by HSF-1 upon HS. *miR-34*, a miRNA induced by HSF-1 upon HS, is predicted to suppress *mdt-15*. In *C*. *elegans*, MDT-15 is an evolutionary conserved subunit of a mediator complex that is required for the SKN-1-mediated oxidative stress response [36]. The downregulation of *mdt-15* by *miR-34* may therefore be one method of suppressing the oxidative stress response during a heat stress. This may be advantageous for heat stress survival, as the oxidative stress response has been shown to impair the HSR [37–39]. We also see that *miR-48*, a miRNA we identified to be suppressed by HSF-1 upon HS, may result in upregulation of *aip-1*, a known SKN-1 and HSF-1 target gene [8, 40]. *Aip-1* is a component of the proteasome that functions to increase the accessibility of a protein substrate to the proteasome, ultimately assisting in adaptation to proteotoxic stressors [41]. Overall, these data suggest that the ability of HSF-1 to control miRNA expression upon heat stress may be one method utilized by organisms to regulate stress-specific gene expression that may confer stress-specific adaptation.

#### Cytoprotection, development, metabolism, and longevity are predicted to be impacted during HS by HSF-1-regulated miRNAs

Next, DAVID was used to determine the globally enriched biological processes impacted by the miRNAs regulated by HSF-1 during HS (S7 Fig). Induction of *miR-784*, *miR-231*, *miR-86*, *miR-53*, *miR-47*, and *miR-34* by HSF-1 during HS is predicted to have the largest impact on the suppression of genes encoding proteins involved in the regulation of transcription and behavior, as these processes have enrichment scores of 5.49 and 4.93, respectively (S7A Fig). Other suppressed processes in this category, with enrichment scores between 4.29–2.7, include the regulation of intracellular signaling, phosphorylation, reproductive behavior, RNA-metabolic processes, cytoskeletal organization, enzyme linked receptor signaling, post-embryonic development, fatty acid metabolic processes, cell death, epithelium development, and aging. These biological processes encompass development, metabolism, and longevity, which are likely the main physiological processes normally suppressed during HS by HSF-1-regulated miRNAs.

We next determined the top biological processes upregulated in response to suppression of *miR-48* and *miR-228* by HSF-1 during HS (S7B Fig). The induction of genes encoding proteins involved in cellular homeostasis and reproductive behavior constitute the top induced processes with enrichment scores of 2.71 and 2.01, respectively. Other induced processes in this category, with enrichment scores between 1.68 and 1.41, include genes encoding proteins involved in cuticle formation and post-embryonic development. These processes can be linked to cytoprotection, thus cytoprotective processes are normally induced by HSF-1-regulated miRNAs during HS.

### Global biological processes impacted independently of HS by HSF-1-regulated miRNAs

#### Insulin-like signaling factors link miRNA/mRNA networks regulated by HSF-1 independently of HS

**Fig 5:**
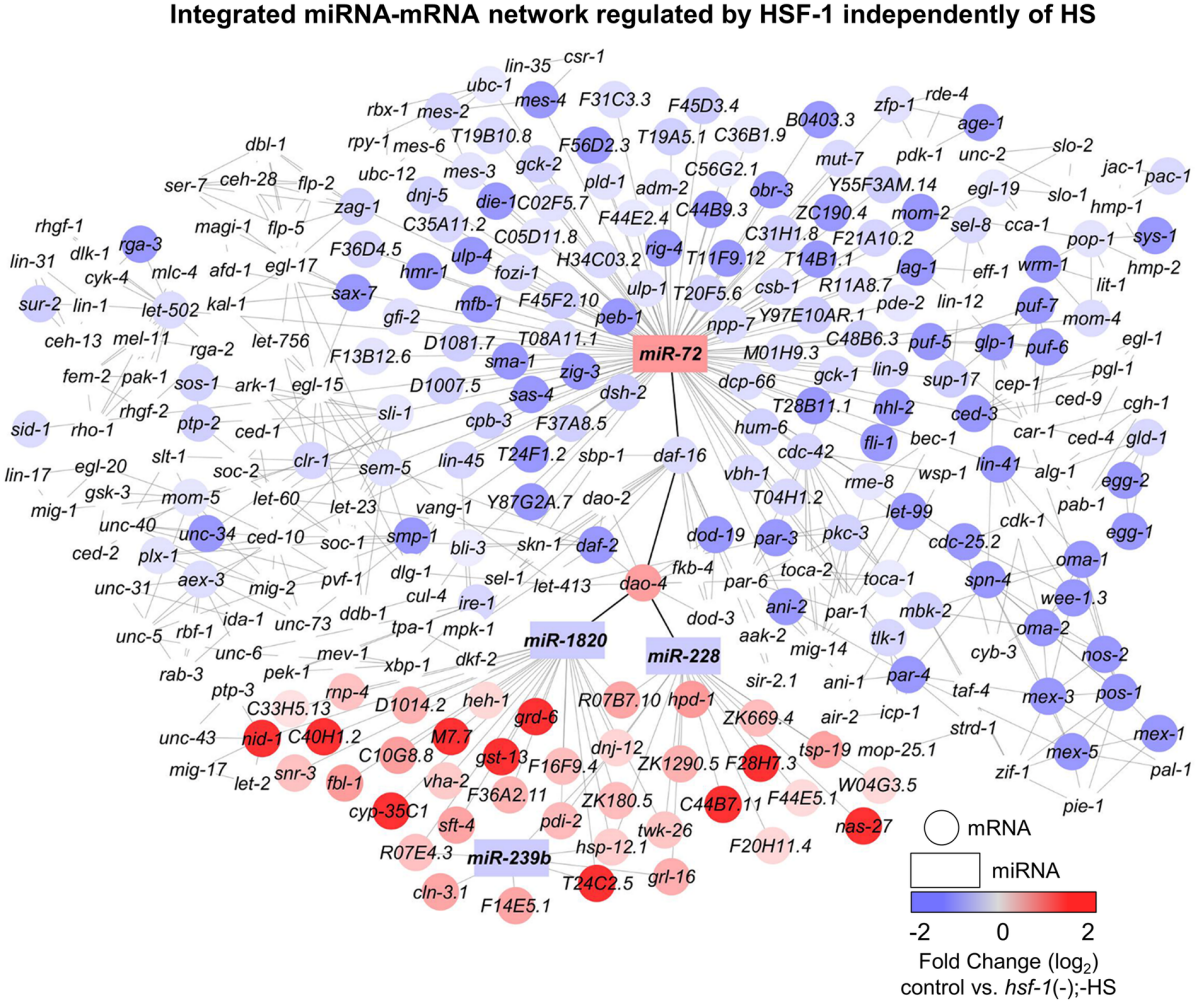
Integrated target prediction analysis uncovers miRNA/mRNA interaction networks regulated by HSF-1 independently of HS. The miRNAs (rectangles) that we determined to be regulated by HSF-1 independently of HS were used for target prediction analysis carried out by mirWIP or TargetScan. The mRNA targets (circles) were consolidated by comparing the predicted mRNAs to those determined by mRNA-sequencing, performed in parallel to miRNA sequencing, to be regulated by HSF-1 independently of HS. Interactions were predicted using the Agilent literature search tool, and network generation was done with Cytoscape. Transcripts that are not colored were not affected in our dataset, but are neighbors shared by at least two transcripts that were affected. The color of each miRNA or mRNA corresponds to the degree of HSF-1 regulation, where red represents induction and blue represents suppression. Bold connecting lines represent connections between upregulated and downregulated clusters.

To determine a broad impact for HSF-1 in post-transcriptionally regulating gene expression independently of HS, we again used the software Cytoscape and the Agilent literature search tool to build an interaction network with the integrated miRNA/mRNA network we determined to be regulated by HSF-1 independently of HS (Fig 5). Predicted mRNA targets were overlaid with the expression changes obtained from mRNA-seq, performed in parallel to this study [7], where blue corresponds to negative regulation and red corresponds to positive regulation by HSF-1 independently of HS. Uncolored genes were not affected in our dataset, but are neighbors shared by at least two genes that were affected. This method of network generation thus allows for the determination of a broad network of genes predicted to be post-transcriptionally regulated by HSF-1 independently of HS.

To uncover important linkages between the induced and suppressed networks, we grouped upregulated and downregulated processes in order to visualize connecting mRNAs (Fig 5, represented as bold connecting lines). Interestingly, we uncovered the insulin-like signaling factors *daf-16* and *dao-4* as links between the induced and suppressed miRNAs regulated by HSF-1 independently of HS. We have found a potential regulatory link between *miR-72*, a miRNA we show to normally be induced by HSF-1 independently of HS, and *daf-16*. Controlling *daf-16* may be one mechanism utilized by HSF-1 to fine-tune metabolism and longevity independently of HS. The *daf-16* target gene *dao-4* is predicted to be upregulated in response to suppression of *miR-1820* and *miR-228* by HSF-1. HSF-1 has previously been linked to having a non-stress role in controlling metabolism, where caloric restriction, metabolic factors, and insulin-like signaling factors have been linked to HSF-1 and the HSR [8, 42–45]. Recent studies suggest that the insulin-like signaling factor and FOXO transcription factor DAF-16 has a stress-independent role in controlling HSF-1-mediated lifespan extension [46]. Overall, these data suggest that HSF-1-regulated miRNA networks may be connected through insulin-like signaling factors to control metabolism and lifespan in a heat-stress independent fashion.

#### Development, metabolism, and longevity are predicted to be impacted by HSF-1-regulated miRNAs independently of HS

Next, DAVID was used to determine the globally enriched biological processes impacted by the miRNAs regulated by HSF-1 independently of HS (S8 Fig). Induction of *miR-72* by HSF-1 is predicted to have the largest impact on suppressing genes encoding proteins involved in the regulation of post-embryonic development and cell migration, as these processes have enrichment scores of 13.68 and 10.14, respectively (S8A Fig). Other suppressed processes in this category, with enrichment scores between 8.28–2.48, include the regulation of phosphorylation, sex differentiation, apoptosis, growth, epithelium development, axis specification, cell adhesion, reproductive development, behavior, aging, neuron development, cuticle development, and RNA metabolic processes. These processes are involved in development, further supporting our previous work suggesting HSF-1 as a global regulator of developmental processes independently of HS [7].

We next determined the biological processes upregulated in response to suppression of *miR-1820* and *miR-228* by HSF-1 independently of HS (S8B Fig). Each upregulated process has a relatively low enrichment score (under 1.02), suggesting that suppression of biological processes is the primary role of HSF-1-regulated miRNAs. However, the most highly upregulated processes are phosphate metabolic processes and growth, with enrichment scores of 1.02 and 0.74, respectively. The other induced process in this category, with an enrichment score of 0.61, is epithelium development. Overall, genes encoding proteins involved in regulating developmental processes may be induced by HSF-1-regulated miRNAs independently of HS.

### HSF-1 may impact longevity through the post-transcriptional control of collagen and cytoskeletal genes

In this study, we show that cuticle processes are enriched by HSF-1 upon HS and suppressed by HSF-1 independently of HS. Collagen genes, which encode cuticle proteins, were recently shown to impact lifespan and to be regulated by HSF-1 during and independently of HS [47]. The oxidative stress factor SKN-1 may also regulate the expression of specific collagen genes to control longevity, supporting a role for collagen genes in regulating longevity during stress [47]. These data suggest a post-transcriptional role for HSF-1 in regulating specific cuticle collagen genes, further supporting our previous work suggesting HSF-1 as a regulator of collagen gene expression. Ultimately, the regulation of collagens may influence aging and longevity during stressors.

Cytoskeletal organization may be another process impacted by HSF-1-regulated miRNAs, and may be one method to promote cytoprotection and longevity during HS. HSF-1 was recently shown to regulate genes that control cytoskeletal stability which extended lifespan and promoted stress resistance in *C*. *elegans* [48]. Thus, these data suggest HSF-1 may post-transcriptionally control cytoskeletal processes and ultimately influence longevity.

## Conclusion

**Fig 6:**
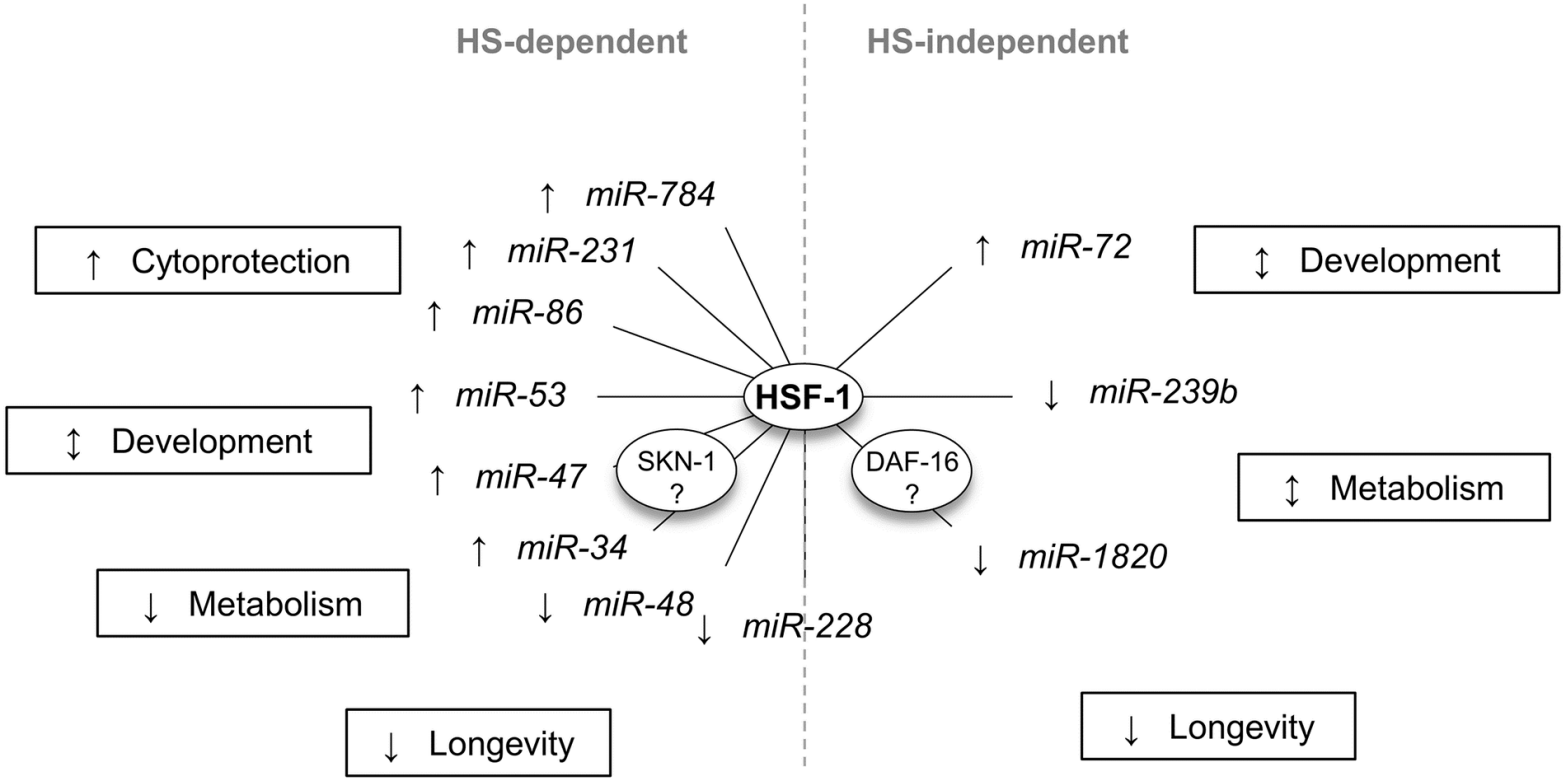
A model for heat stress-dependent and -independent processes controlled by HSF-1-regulated miRNAs. HSF-1 controls miRNA expression during and independently of HS. During HS, HSF-1 is predicted to post-transcriptionally regulate genes involved in cytoprotection, development, metabolism, and longevity. These processes may be connected through the oxidative stress response transcription factor SKN-1. Independently of HS, HSF-1 is predicted to post-transcriptionally regulate genes involved in development, metabolism, and longevity. These processes may be connected through the insulin-like signaling transcription factor DAF-16. This work highlights a possible role for HSF-1 in post-transcriptionally regulating gene expression and various biological processes, and provides a possible mechanism for cross-talk between stress responses.

The miRNA-sequencing experiment performed in this study has allowed us to uncover a possible post-transcriptional role for HSF-1 in regulating heat stress-dependent and -independent processes in *C*. *elegans* (Fig 6). During HS, HSF-1 is predicted to post-transcriptionally promote specific genes encoding proteins involved in cytoprotection and development, while suppressing other genes encoding proteins involved in development, metabolism, and longevity. Independently of HS, HSF-1 is predicted to post-transcriptionally promote specific genes encoding proteins involved in development and metabolism, while suppressing other genes encoding proteins involved in development, metabolism, and longevity. Integrated miRNA/mRNA network analyses point to HSF-1-regulated miRNAs as links between the oxidative stress response and the insulin-like signaling pathway to HSF-1-regulated processes, suggesting a mechanism for cross-talk between stress responses. Additionally, this work further highlights a role for HSF-1 in regulating the expression of cuticle collagen genes which may control longevity. Overall, our work has uncovered a potential role for HSF-1 in post-transcriptionally controlling gene expression in *C*. *elegans*, and suggests a mechanism for cross-talk between stress responses.

## Supporting information

S1 Fig Validation of RNAi treatment conditions for miRNA-seq experiments. (**A**) *hsf-1* RNAi blunts *hsp-70* promoter activity upon HS. Fluorescent images are shown of synchronous p*C12C8*.*1*(*hsp-70*)::GFP worms fed controla RNAi [*hsf-1*(+)] or *hsf-1* RNAi [*hsf-1*(-)] from the L1 larval stage to the L4 larval stage prior to treatment with a 33°C 30 minute heat shock (+HS) followed by a 12 hour recovery. (**B**) Quantification of fluorescence intensity confirms that *hsf-1* RNAi blunts *hsp-70* promoter activity upon HS. Quantification of the fluorescent images in (**A**) demonstrate *hsf-1* RNAi decreases *hsp-70* promoter activity by ~80%. (**C**) *hsf-1* RNAi blunts *hsp-16*.*2* promoter activity upon HS. Fluorescent images are shown of synchronous p*Y46H3A*.*3*(*hsp-16*.*2*)::GFP worms fed control RNAi [*hsf-1*(+)] or *hsf-1* RNAi [*hsf-1*(-)] from the L1 larval stage to the L4 larval stage prior to treatment with a 33°C 30 minute heat shock (+HS) followed by a 12 hour recovery. (**D**) Quantification of fluorescence intensity confirms that *hsf-1* RNAi blunts *hsp-16*.*2* promoter activity upon HS. Quantification of the fluorescent images in (**C**) demonstrate *hsf-1* RNAi decreases *hsp-16*.*2* promoter activity ~60%. (**E**) Heat shock protein genes are the top genes induced during a 30 minute 33°C HS. mRNA-seq performed in parallel to miRNA-seq [7] shows that *hsp* genes are the highest induced group of genes in response to HS, and shows these genes are dependent on HSF-1, further validating our experimental conditions. For (**B,D**), error bars represent standard deviation and significance was determined with the Bonferroni post-test where ‘***’;q-value<0.001.
(TIF)

S2 Fig Dendogram analysis and differential expression between miRNA-seq biological duplicates shows a correlation between biological replicates. (**A**) Dendogram correlation between biological duplicates. Clustering of the biological duplicates for each miRNA-seq condition reveals conserved alignment between replicates. The dendogram was generated by the program CummeRbund to provide insight into the relationships between different conditions. (**B-E**) Differential expression analysis shows little deviation between biological duplicates. Scatter plots of the miRNA-seq reads for each biological replicate, for each condition, shows similarities between biological duplicates. The x-axis is representative of the reads from the first biological replicate, and the y-axis represents reads from the second biological replicate. The closer the R^2^ value is to 1, and the closer each point is to the line, is representative of similarity between replicates.
(TIF)

S3 Fig Volcano plots for each miRNA-seq condition relative to the control. Volcano plots show all unchanged (q-value>0.05) and significantly altered (q-value<0.05) miRNAs, relative to the *hsf-1*(+);-HS control. The q-value is the FDR-adjusted p-value of the test statistic as determined by the Benjamini-Hochberg correction for multiple testing.
(TIF)

S4 Fig miRNAs normally upregulated by HSF-1 during HS. (**A**) Venn diagram analysis of the miRNAs significantly upregulated as compared to the control. The Venn diagram shows the overlap among miRNAs that were found to be significantly upregulated (q-value<0.05) as compared to the *hsf-1*(+);-HS control for each of the indicated comparisons between samples. The blue shaded region represents miRNAs that are regulated by *hsf-1* during HS. The q-value is the FDR-adjusted p-value of the test statistic as determined by the Benjamini-Hochberg correction for multiple testing (**B**) Relative abundance of the miRNAs normally upregulated by HSF-1 upon HS. The log_2_ fold change from the miRNA-seq data, of the miRNAs determined via Venn diagram analysis to be regulated by HSF-1 upon HS as compared to the *hsf-1*(+);-HS control, shows the miRNAs determined to be significantly different compared to each treatment condition. Significance was determined with the Benjamini-Hochberg correction where ‘*’;q-value<0.05. (**C**) miRNAs determined to normally be upregulated by HSF-1 during HS. The miRNAs that had a significant difference between the *hsf-1*(+);+HS and *hsf-1*(-);+HS treatment conditions in (B), as determined by the Benjamini-Hochberg correction for multiple testing, are listed.
(TIF)

S5 Fig miRNAs normally downregulated by HSF-1 during HS. (**A**) Venn diagram of the miRNAs significantly downregulated compared to the control. The Venn diagram shows the overlap among miRNAs that were found to be significantly downregulated (q-value<0.05) as compared to the *hsf-1*(+);-HS control for each of the indicated comparisons between samples. The blue shaded region represents miRNAs that are normally downregulated by *hsf-1* during HS. The q-value is the FDR-adjusted p-value of the test statistic as determined by the Benjamini-Hochberg correction for multiple testing (**B**) Relative abundance of the miRNAs normally downregulated by HSF-1 upon HS. The log_2_ fold change from the miRNA-seq data, of the miRNAs determined via Venn diagram analysis to be regulated by HSF-1 upon HS as compared to the *hsf-1*(+);-HS control, shows the miRNAs determined to be significantly different compared to each treatment condition. Significance was determined with the Benjamini-Hochberg correction where ‘*’;q-value<0.05. (**C**) miRNAs determined to normally be downregulated by HSF-1 during HS. The miRNAs that had a significant difference between the *hsf-1*(+);+HS and *hsf-1*(-);+HS treatment conditions in (**B**) as determined by the Benjamini-Hochberg correction for multiple testing are listed.
(TIF)

S6 Fig miRNAs normally regulated by HSF-1 independently of HS. (**A**) Venn diagram analysis of the miRNAs significantly upregulated as compared to the control. The Venn diagram shows the overlap among miRNAs that were found to be significantly downregulated (q-value<0.05) as compared to the *hsf-1*(+);-HS control for each of the indicated comparisons between samples. A miRNA that is downregulated in response to *hsf-1* RNAi is considered to normally be upregulated by HSF-1. The yellow shaded region thus represents miRNAs that are normally upregulated by *hsf-1* independently of HS. The q-value is the FDR-adjusted p-value of the test statistic as determined by the Benjamini-Hochberg correction for multiple testing (**B**) Relative abundance of the miRNAs normally upregulated by HSF-1 independently of HS. The log_2_ fold change from the miRNA-seq data, of the miRNAs determined via Venn diagram analysis to be regulated by HSF-1 independently of HS as compared to the *hsf-1*(+);-HS control, shows the miRNAs determined to be significantly different compared to each treatment condition. Significance was determined with the Benjamini-Hochberg correction where ‘*’;q-value<0.05. (**C**) miRNAs determined to normally be upregulated by HSF-1 independently of HS. The miRNAs that did not have a significant difference between the *hsf-1*(-);+HS and *hsf-1*(-);-HS treatment conditions in (**B**), as determined by the Benjamini-Hochberg correction for multiple testing, are listed. (**D**) Venn diagram analysis of the miRNAs significantly downregulated as compared to the control. The Venn diagram analysis shows the overlap among miRNAs that were found to be significantly upregulated (q-value<0.05) as compared to the *hsf-1*(+);-HS control for each of the indicated comparisons between samples. A miRNA that is upregulated in response to *hsf-1* RNAi is considered to normally be downregulated by HSF-1. The yellow shaded region thus represents miRNAs that are normally downregulated by *hsf-1* independently of HS. The q-value is the FDR-adjusted p-value of the test statistic as determined by the Benjamini-Hochberg correction for multiple testing (**E**) Relative abundance of the miRNAs normally downregulated by HSF-1 independently of HS. The log_2_ fold change from the miRNA-seq data, of the miRNAs determined via Venn diagram analysis to be regulated by HSF-1 independently of HS as compared to the *hsf-1*(+);-HS control, shows the miRNAs determined to be significantly different compared to each treatment condition. Significance was determined with the Benjamini-Hochberg correction where ‘*’;q-value<0.05. (**F**) miRNAs determined to normally be downregulated by HSF-1 independently of HS. The miRNAs that did not have a significant difference between the *hsf-1*(+);+HS and *hsf-1*(-);+HS treatment conditions in (**E**), as determined by the Benjamini-Hochberg correction for multiple testing, are listed.
(TIF)

S7 Fig Biological processes enriched by HSF-1-regulated miRNAs during HS. (**A**) Processes normally downregulated by HSF-1-regulated miRNAs during HS. The genes predicted to be suppressed by HSF-1-regulated miRNAs during HS were classified by Gene Ontology terms using DAVID. Processes with an enrichment score>~2.5 are listed. (**B**) Processes normally upregulated by HSF-1-regulated miRNAs during HS. The genes predicted to be induced by HSF-1-regulated miRNAs during HS were classified by Gene Ontology terms using DAVID. The top 4 processes are listed.
(TIF)

S8 Fig Biological processes enriched by HSF-1-regulated miRNAs independently of HS. (**A**) Processes normally downregulated by HSF-1-regulated miRNAs independently of HS. The genes predicted to be suppressed by HSF-1-regulated miRNAs independently of HS were classified by Gene Ontology terms using DAVID. Processes with an enrichment score>~2.48 are listed. (**B**) Processes normally upregulated by HSF-1-regulated miRNAs independently of HS. The genes predicted to be induced by HSF-1-regulated miRNAs during HS were classified by Gene Ontology terms using DAVID. The top 3 processes are listed.
(TIF)

S1 Table Abundance of the significantly altered miRNAs for each experimental condition relative to the *hsf-1*(+);-HS control. (XLSX)

S2 Table List of miRNAs unique to each section of the Venn diagram. (XLSX)

S3 Table List of miRNAs determined to be regulated by HSF-1 during and independently of HS. (XLSX)
