# Supplementary figures and images for: HSF-1 is a regulator of miRNA expression in Caenorhabditis elegans
Source: PLoS One. 2017 Aug 24;12(8):e0183445. doi: 10.1371/journal.pone.0183445 (PMC5570370; doi:10.1371/journal.pone.0183445)

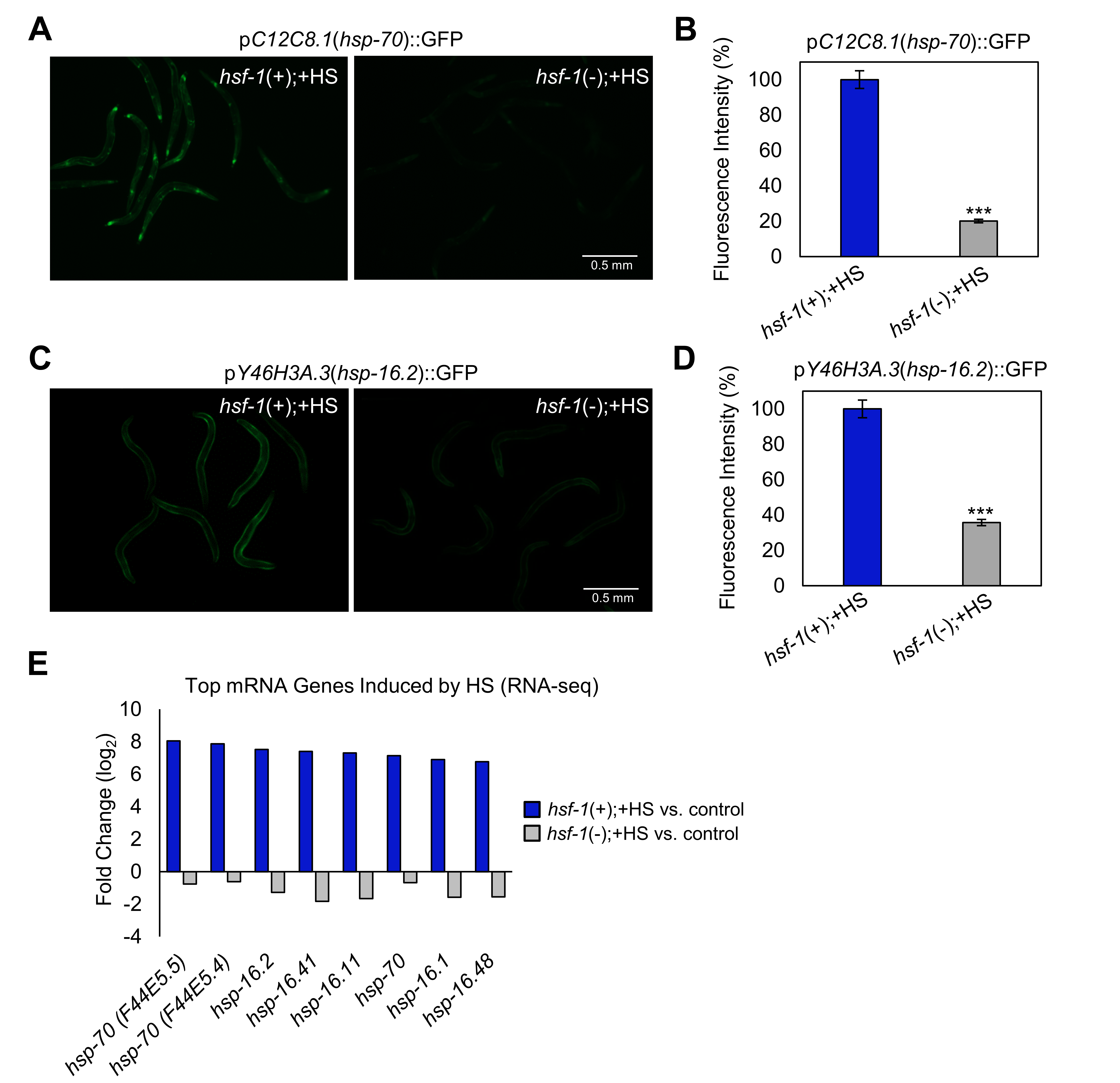

Supplement: S1 Fig — (A) hsf-1 RNAi blunts hsp-70 promoter activity upon HS. Fluorescent images are shown of synchronous pC12C8.1(hsp-70)::GFP worms fed controla RNAi [hsf-1(+)] or hsf-1 RNAi [hsf-1(-)] from the L1 larval stage to the L4 larval stage prior to treatment with a 33°C 30 minute heat shock (+HS) followed by a 12 hour recovery. (B) Quantification of fluorescence intensity confirms that hsf-1 RNAi blunts hsp-70 promoter activity upon HS. Quantification of the fluorescent images in (A) demonstrate hsf-1 RNAi decreases hsp-70 promoter activity by ~80%. (C) hsf-1 RNAi blunts hsp-16.2 promoter activity upon HS. Fluorescent images are shown of synchronous pY46H3A.3(hsp-16.2)::GFP worms fed control RNAi [hsf-1(+)] or hsf-1 RNAi [hsf-1(-)] from the L1 larval stage to the L4 larval stage prior to treatment with a 33°C 30 minute heat shock (+HS) followed by a 12 hour recovery. (D) Quantification of fluorescence intensity confirms that hsf-1 RNAi blunts hsp-16.2 promoter activity upon HS. Quantification of the fluorescent images in (C) demonstrate hsf-1 RNAi decreases hsp-16.2 promoter activity ~60%. (E) Heat shock protein genes are the top genes induced during a 30 minute 33°C HS. mRNA-seq performed in parallel to miRNA-seq [7] shows that hsp genes are the highest induced group of genes in response to HS, and shows these genes are dependent on HSF-1, further validating our experimental conditions. For (B,D), error bars represent standard deviation and significance was determined with the Bonferroni post-test where ‘***’;q-value<0.001. (TIF) [file pone.0183445.s001.TIF]

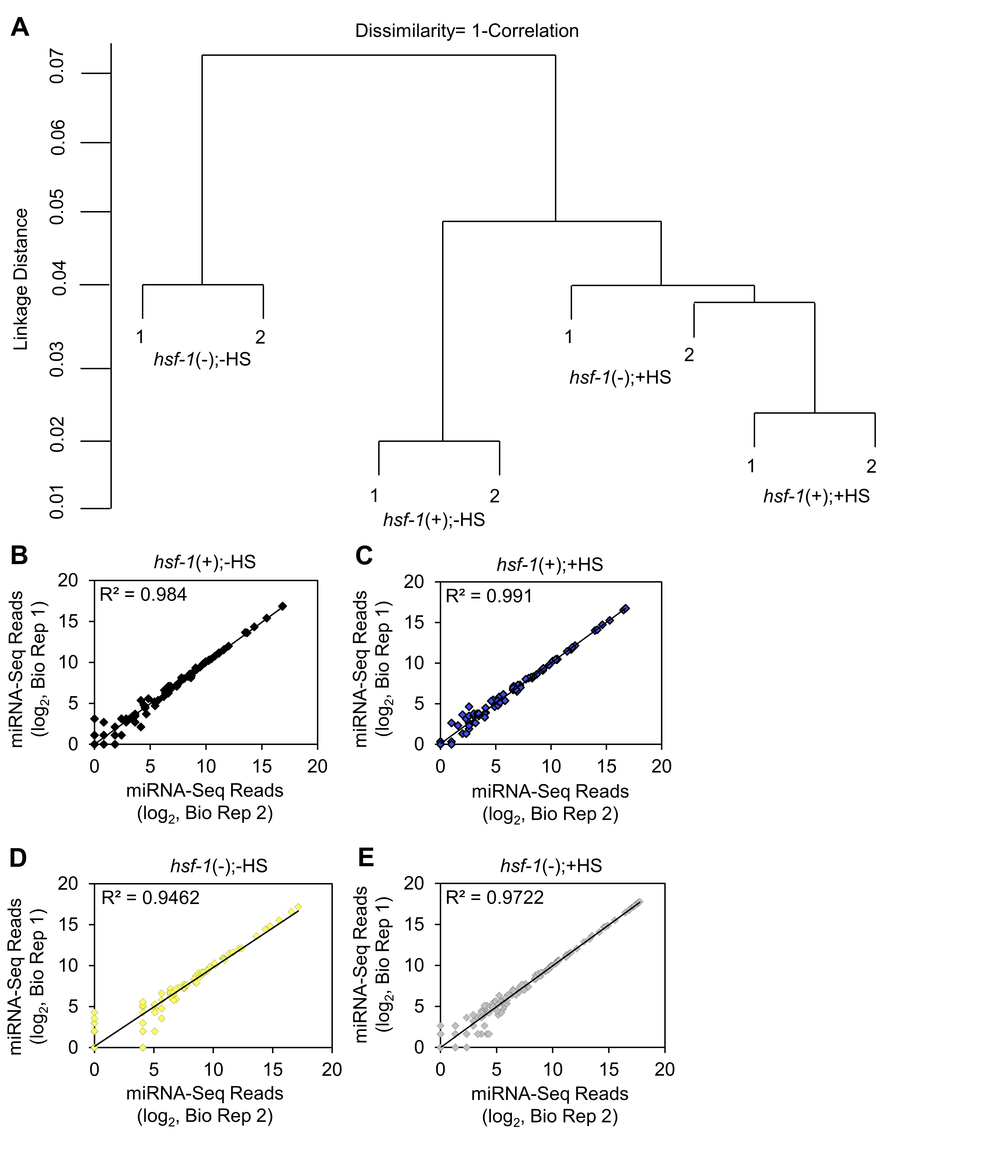

Supplement: S2 Fig — (A) Dendogram correlation between biological duplicates. Clustering of the biological duplicates for each miRNA-seq condition reveals conserved alignment between replicates. The dendogram was generated by the program CummeRbund to provide insight into the relationships between different conditions. (B-E) Differential expression analysis shows little deviation between biological duplicates. Scatter plots of the miRNA-seq reads for each biological replicate, for each condition, shows similarities between biological duplicates. The x-axis is representative of the reads from the first biological replicate, and the y-axis represents reads from the second biological replicate. The closer the R2 value is to 1, and the closer each point is to the line, is representative of similarity between replicates. (TIF) [file pone.0183445.s002.TIF]

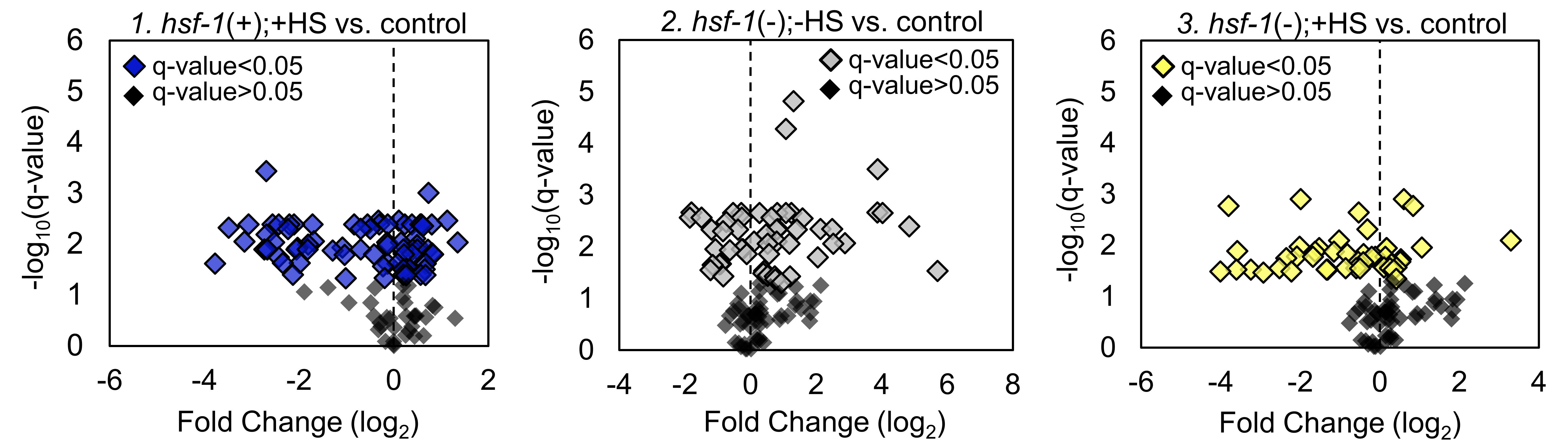

Supplement: S3 Fig — Volcano plots show all unchanged (q-value>0.05) and significantly altered (q-value<0.05) miRNAs, relative to the hsf-1(+);-HS control. The q-value is the FDR-adjusted p-value of the test statistic as determined by the Benjamini-Hochberg correction for multiple testing. (TIF) [file pone.0183445.s003.TIF]

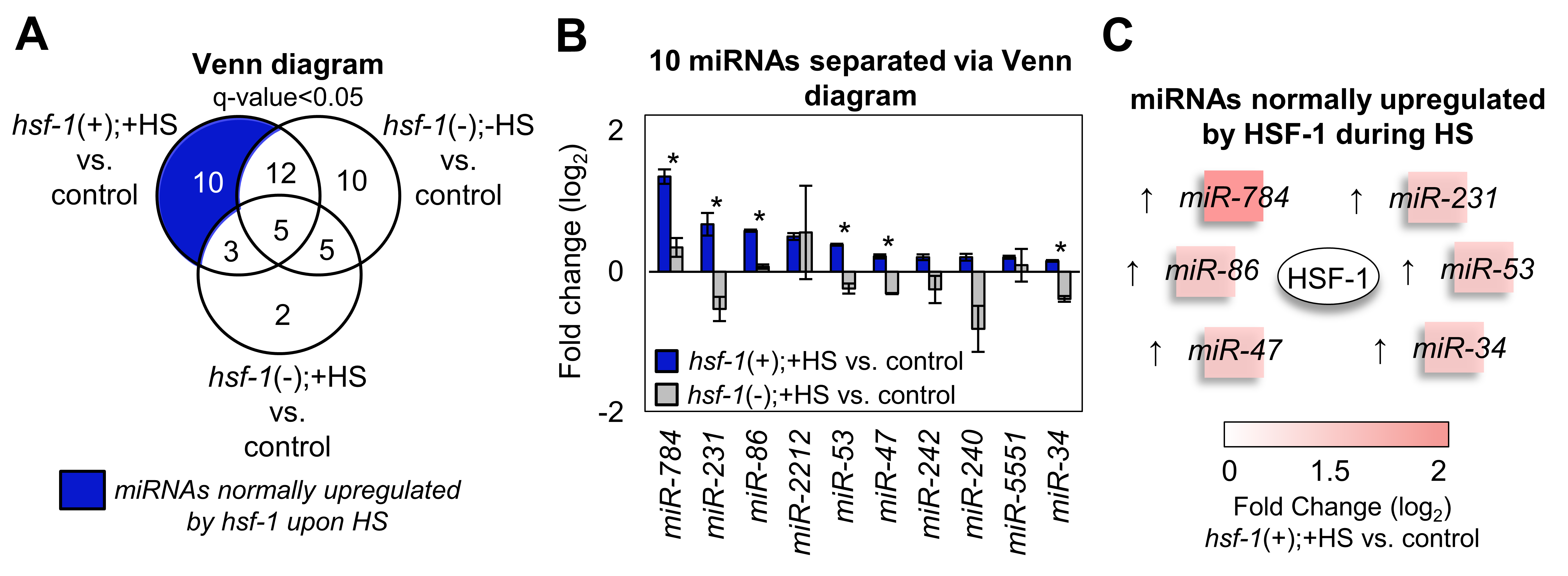

Supplement: S4 Fig — (A) Venn diagram analysis of the miRNAs significantly upregulated as compared to the control. The Venn diagram shows the overlap among miRNAs that were found to be significantly upregulated (q-value<0.05) as compared to the hsf-1(+);-HS control for each of the indicated comparisons between samples. The blue shaded region represents miRNAs that are regulated by hsf-1 during HS. The q-value is the FDR-adjusted p-value of the test statistic as determined by the Benjamini-Hochberg correction for multiple testing (B) Relative abundance of the miRNAs normally upregulated by HSF-1 upon HS. The log2 fold change from the miRNA-seq data, of the miRNAs determined via Venn diagram analysis to be regulated by HSF-1 upon HS as compared to the hsf-1(+);-HS control, shows the miRNAs determined to be significantly different compared to each treatment condition. Significance was determined with the Benjamini-Hochberg correction where ‘*’;q-value<0.05. (C) miRNAs determined to normally be upregulated by HSF-1 during HS. The miRNAs that had a significant difference between the hsf-1(+);+HS and hsf-1(-);+HS treatment conditions in (B), as determined by the Benjamini-Hochberg correction for multiple testing, are listed. (TIF) [file pone.0183445.s004.TIF]

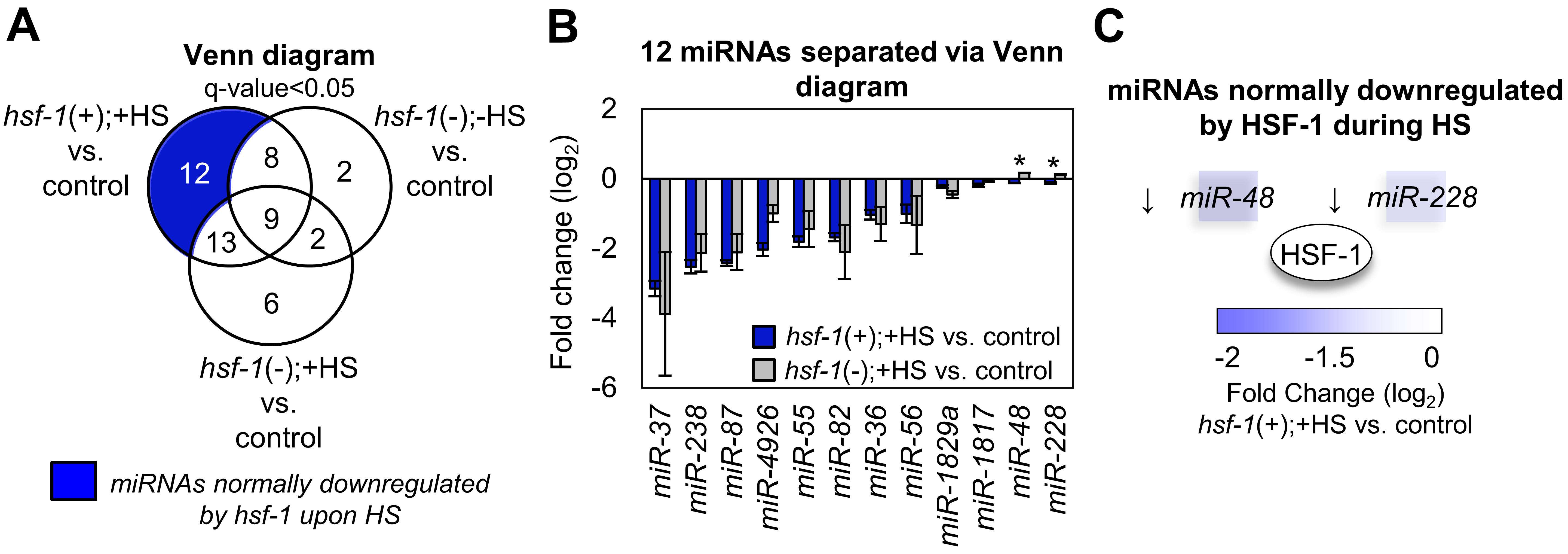

Supplement: S5 Fig — (A) Venn diagram of the miRNAs significantly downregulated compared to the control. The Venn diagram shows the overlap among miRNAs that were found to be significantly downregulated (q-value<0.05) as compared to the hsf-1(+);-HS control for each of the indicated comparisons between samples. The blue shaded region represents miRNAs that are normally downregulated by hsf-1 during HS. The q-value is the FDR-adjusted p-value of the test statistic as determined by the Benjamini-Hochberg correction for multiple testing (B) Relative abundance of the miRNAs normally downregulated by HSF-1 upon HS. The log2 fold change from the miRNA-seq data, of the miRNAs determined via Venn diagram analysis to be regulated by HSF-1 upon HS as compared to the hsf-1(+);-HS control, shows the miRNAs determined to be significantly different compared to each treatment condition. Significance was determined with the Benjamini-Hochberg correction where ‘*’;q-value<0.05. (C) miRNAs determined to normally be downregulated by HSF-1 during HS. The miRNAs that had a significant difference between the hsf-1(+);+HS and hsf-1(-);+HS treatment conditions in (B) as determined by the Benjamini-Hochberg correction for multiple testing are listed. (TIF) [file pone.0183445.s005.TIF]

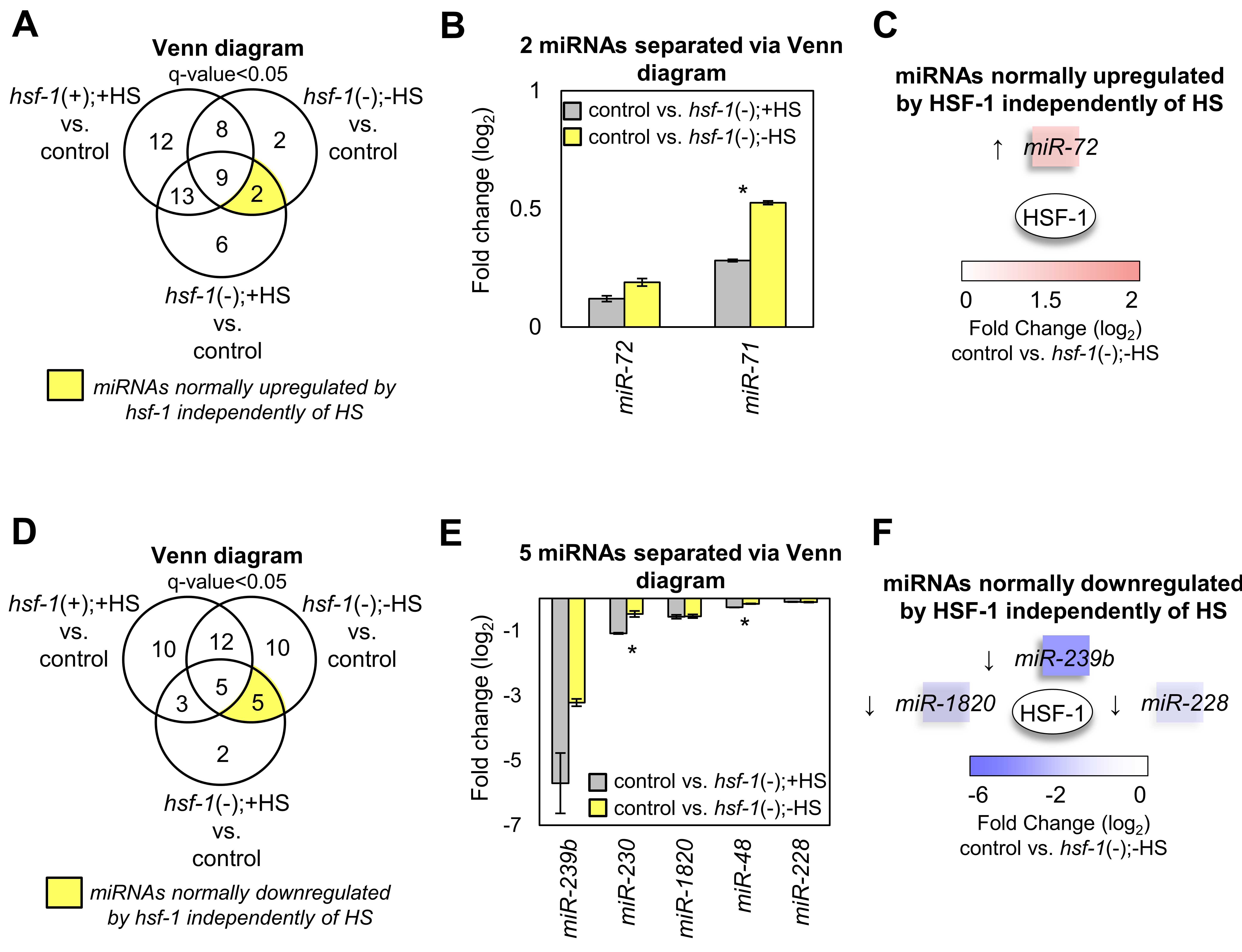

Supplement: S6 Fig — (A) Venn diagram analysis of the miRNAs significantly upregulated as compared to the control. The Venn diagram shows the overlap among miRNAs that were found to be significantly downregulated (q-value<0.05) as compared to the hsf-1(+);-HS control for each of the indicated comparisons between samples. A miRNA that is downregulated in response to hsf-1 RNAi is considered to normally be upregulated by HSF-1. The yellow shaded region thus represents miRNAs that are normally upregulated by hsf-1 independently of HS. The q-value is the FDR-adjusted p-value of the test statistic as determined by the Benjamini-Hochberg correction for multiple testing (B) Relative abundance of the miRNAs normally upregulated by HSF-1 independently of HS. The log2 fold change from the miRNA-seq data, of the miRNAs determined via Venn diagram analysis to be regulated by HSF-1 independently of HS as compared to the hsf-1(+);-HS control, shows the miRNAs determined to be significantly different compared to each treatment condition. Significance was determined with the Benjamini-Hochberg correction where ‘*’;q-value<0.05. (C) miRNAs determined to normally be upregulated by HSF-1 independently of HS. The miRNAs that did not have a significant difference between the hsf-1(-);+HS and hsf-1(-);-HS treatment conditions in (B), as determined by the Benjamini-Hochberg correction for multiple testing, are listed. (D) Venn diagram analysis of the miRNAs significantly downregulated as compared to the control. The Venn diagram analysis shows the overlap among miRNAs that were found to be significantly upregulated (q-value<0.05) as compared to the hsf-1(+);-HS control for each of the indicated comparisons between samples. A miRNA that is upregulated in response to hsf-1 RNAi is considered to normally be downregulated by HSF-1. The yellow shaded region thus represents miRNAs that are normally downregulated by hsf-1 independently of HS. The q-value is the FDR-adjusted p-value of the test statistic as determine [file pone.0183445.s006.tif]

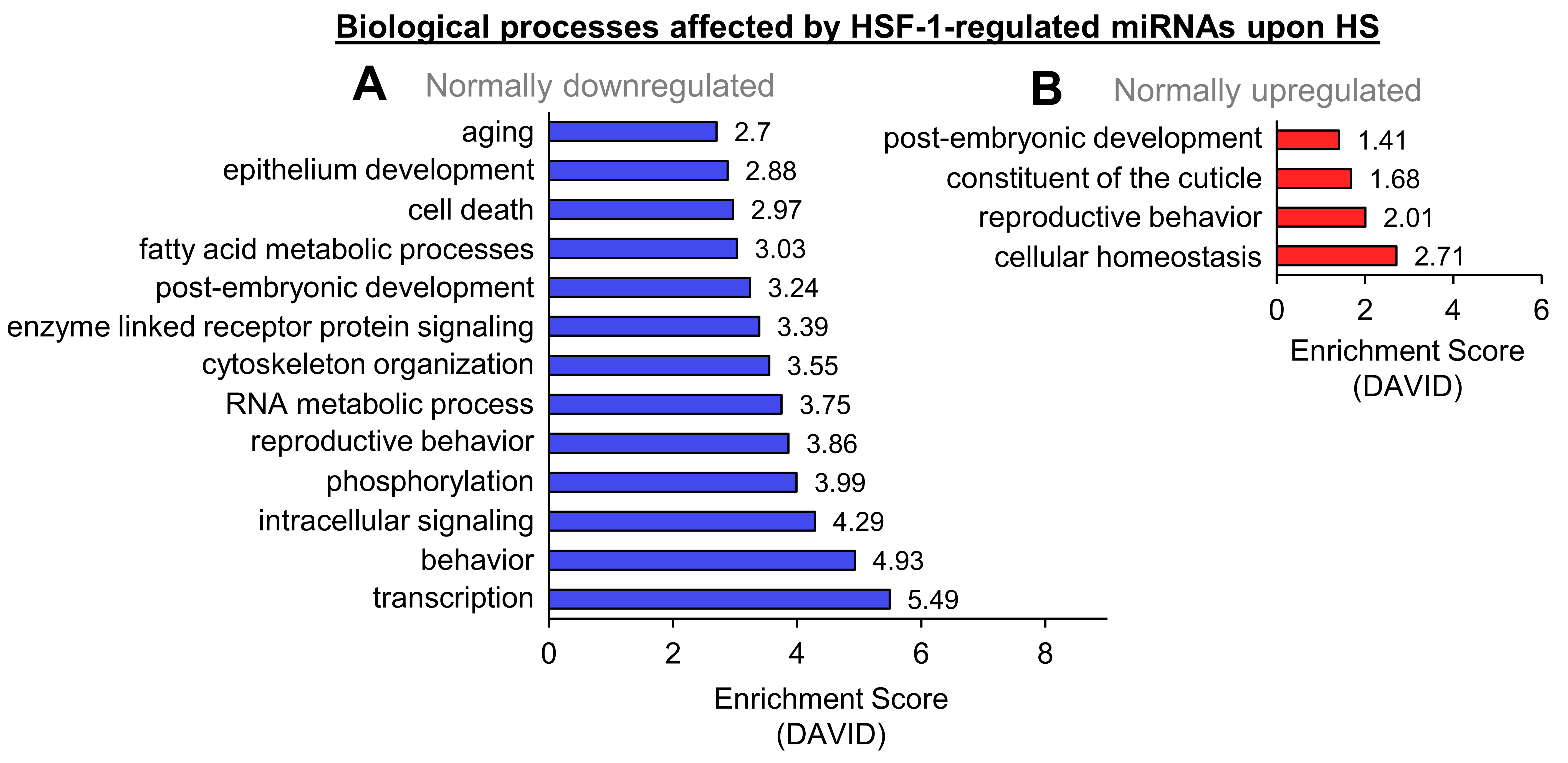

Supplement: S7 Fig — (A) Processes normally downregulated by HSF-1-regulated miRNAs during HS. The genes predicted to be suppressed by HSF-1-regulated miRNAs during HS were classified by Gene Ontology terms using DAVID. Processes with an enrichment score>~2.5 are listed. (B) Processes normally upregulated by HSF-1-regulated miRNAs during HS. The genes predicted to be induced by HSF-1-regulated miRNAs during HS were classified by Gene Ontology terms using DAVID. The top 4 processes are listed. (TIF) [file pone.0183445.s007.TIF]

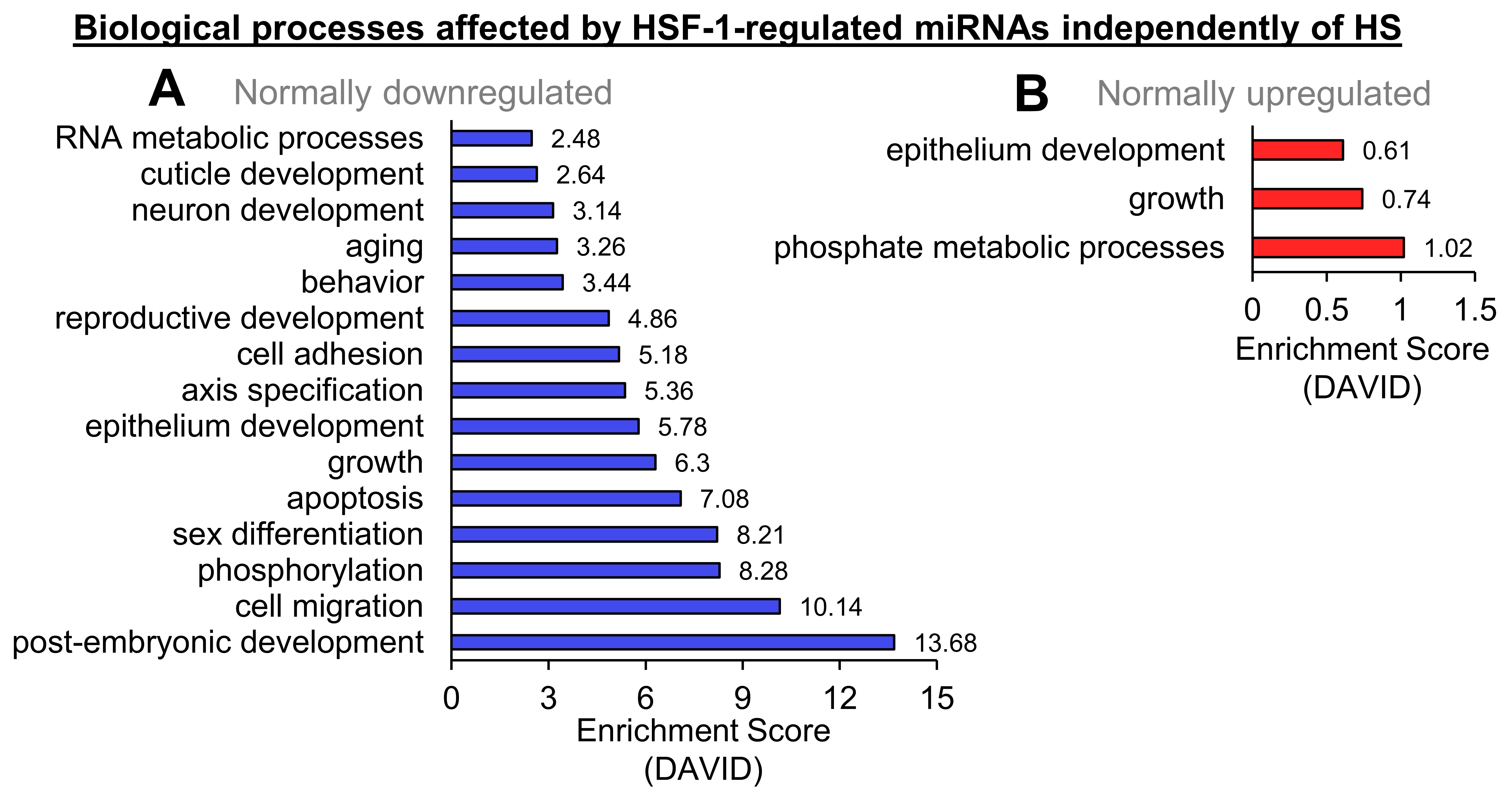

Supplement: S8 Fig — (A) Processes normally downregulated by HSF-1-regulated miRNAs independently of HS. The genes predicted to be suppressed by HSF-1-regulated miRNAs independently of HS were classified by Gene Ontology terms using DAVID. Processes with an enrichment score>~2.48 are listed. (B) Processes normally upregulated by HSF-1-regulated miRNAs independently of HS. The genes predicted to be induced by HSF-1-regulated miRNAs during HS were classified by Gene Ontology terms using DAVID. The top 3 processes are listed. (TIF) [file pone.0183445.s008.TIF]
